# Suppression of Presymptomatic Oxidative Stress and Inflammation in Neurodegeneration by Grape-Derived Polyphenols

**DOI:** 10.3389/fphar.2018.00867

**Published:** 2018-08-28

**Authors:** Francis Herman, Susan Westfall, Justin Brathwaite, Giulio M. Pasinetti

**Affiliations:** ^1^Department of Neurology, Mount Sinai School of Medicine, New York, NY, United States; ^2^Department of Genomic Sciences, Mount Sinai School of Medicine, New York, NY, United States; ^3^James J. Peters VA Medical Center, Bronx, NY, United States

**Keywords:** polyphenols, inflammation, inflammasome, reactive oxygen species, neurodegeneration

## Abstract

Neurodegenerative disorders constitute a group of multifaceted conditions characterized by the progressive loss of neurons and synaptic connections consequent to a combination of specific genetic predispositions and stochastic stressors. The neuropathologies observed in both Alzheimer’s and Parkinson’s disease are in part attributed to compounding intrinsic and extrinsic environmental stressors, which we propose may be limited by the administration of specific grape derived phytochemicals and their metabolized derivatives, specifically polyphenols isolated from grape botanicals. Current therapies for neurodegenerative disorders are limited as they solely target the final disease pathologies including behavioral changes, cognitive deficits, proteinopathies and neuronal loss; however, this strategy is not a sustainable approach toward managing disease onset or progression. This review discusses the application of grape derived polyphenols as an adjunctive treatment paradigm for the prevention of neuropathologies associated with Alzheimer’s disease, Parkinson’s disease and Chronic Traumatic Encephalopathy by simultaneously ameliorating two stochastic stressors that facilitate their disease pathologies: inflammation and oxidative stress. The biophysical attributes of grape-derived polyphenols buffer against redox potential dependent peripheral and neuroinflammation and down regulate the activation of inflammasomes in microglia and astrocytes, which could provide a novel mechanism through which grape-derived polyphenols simultaneously suppress risk factors across pathologically distinct neurodegenerative conditions. This approach therefore offers a prophylactic mode, not feasible through current pharmacological agents, to target activity dependent risk factors for neurodegenerative disorders that manifest over an individual’s lifetime.

## Introduction

Neurodegeneration is the defining feature of a group of age-related neurological disorders characterized by the progressive atrophy of neurons in cortical and subcortical brain structures. The distinct etiological roots of neurodegeneration remain to be fully characterized; however, stochastic environmental stressors and toxins effectuating persistent inflammation and elevated oxidative stress increase the likelihood of developing neurodegenerative conditions and precede canonical neuropathologies commonly associated with neurodegeneration (Verdile et al., 2015). Identifying toxins and stressors responsible for altering homeostatic processes has been an imperative of research efforts, and has measurably strengthened our understanding of how neurodegeneration develops in certain environmental settings. To patients and clinicians, identification of toxins and stressors also supports the development of novel therapeutic regimes that target the oxidative and inflammatory effectors to such environmental exposures. Neurodegeneration is a common condition manifesting as different diseases depending on both genetic and pathological predispositions. Amyotrophic Lateral Sclerosis and Huntington’s disease are neurodegenerative disorders that have strong familial and genetic predispositions (Al-Chalabi et al., 2016), whereas Alzheimer’s disease (AD) and Parkinson’s disease (PD) do have smaller genetic components but are more commonly attributed to stochastic environmental stressors that accumulate over a lifetime (Przedborski et al., 2003). Currently approved therapies for both types of neurodegenerative disorders, focus on alleviating final pathological manifestations or cognitive impairments including decreased working memory, loss of cognitive function, dysregulation of neurotransmitter signaling and the accumulation of protein inclusions commonly observed in neurodegenerative disorders. However, such therapeutic approaches have limitations to their efficacy as they only treat disease symptoms, rather than the effectors of disease pathology. Patients also develop tolerance, necessitating titration with higher doses and incurring both increased economic costs and side effects. Moving toward the development of sustainable and long-term treatment options that effectively ameliorate the disease pathologies, novel therapies should strive to target fundamental mechanisms of the neurodegenerative pathologies. In the context of AD and PD, the accumulation of inflammation and oxidative stress throughout an individual’s lifetime are among the primary aggravators of disease onset and progression (Umeno et al., 2017). Targeting these two mutually aggravating presymptomatic physical states could therefore expand the treatment window that is limited for traditional pharmacological therapies. This review will therefore present evidence supporting the claim that polyphenols, by simultaneously alleviating oxidative stress and inflammasome-mediated inflammation, may fill a unique niche for treatment of presymptomatic neurodegenerative disorders that targeted pharmacological therapies are not designed to provide.

### Alzheimer’s Disease

Alzheimer’s Disease is an age-related neurodegenerative disease with treatment options limited to cholinesterase inhibitors for memory impairments. In later disease stages, AD incurs greater costs due to patient care in handling the progressive memory loss, nervousness, cognitive disability, and the eventual loss of bodily functions that ultimately lead to death. AD is the sixth leading cause of death in the United States affecting 5.4 million Americans and costing the healthcare system an estimated $236 billion: numbers which are expected to triple over the next 30 years (Alzheimer’s Association, 2016). Globally, AD impacts an estimated 46.8 million people costing a sum of $818 billion (Saijo et al., 2010). Between 5–10% of all AD cases can be traced to autosomal dominant mutations that predispose individuals to early onset development (Frisardi et al., 2010); these include mutations in the amyloid precursor protein (APP) and presenilins (PSEN) 1 and 2 (Van Cauwenberghe et al., 2016). The PSEN1 and 2 encode the gamma secretase catalytic unit responsible for producing amyloid beta isoforms from the APP precursor protein. Mutations in either APP or PSEN1/2 lead to the elevated production of the aggregating neurotoxic amyloid beta (Aβ) (1–42) isoform (Klein et al., 1999). It is believed that the overproduction of Aβ42 and amyloid fibrillogenesis contribute in some manner to the cascade of neuropathologies culminating in neurodegeneration characteristic of AD; the extent to which Aβ42 contributes to AD progression is under considerable debate, as anti-Aβ therapies failed to prevent AD-associated cognitive decline, in spite of attenuated brain Aβ depositions.

The remaining sporadic forms of AD are in part attributed to synergistic interplays between the accumulation of lifestyle dependent environmental stressors and genetic polymorphisms prevalent in patients with sporadic AD (Solfrizzi et al., 2004; Gatz et al., 2006; Verdile et al., 2015). Exposure to environmental toxins from smoking or alcohol notably increase the risk of developing AD (O’Brien and Markus, 2014). Additionally, several studies have shown a positive association between diabetes mellitus (Mittal et al., 2016), obesity (Pistollato et al., 2018) and cardiovascular disease (Cermakova et al., 2015) with AD. Altered sleep dynamics also serve as a potent risk factor for the development of AD symptomology (Benedict et al., 2015). Together each of the risk factors associated with AD have the similar basis in that they elevate markers for oxidative stress and inflammation. An individual’s susceptibility to develop AD involves the interactions between these lifestyle factors and various genetic predispositions known to facilitate late-onset AD. Genetic risk loci associated with late-onset AD include the less-favorable allele of apolipoprotein E (APOE), APOE𝜖4 (Michaelson, 2014), which compromises the ability of APOE to promote clearance of Aβ in conjunction with the insulin degrading enzyme (IDE), therefore encouraging Aβ aggregation in the extracellular region (Kim et al., 2009). As an indication of the contribution by stochastic stressors, APOE𝜖4 alone does not necessarily lead to the development of AD as at least 75% of patients heterozygous for APOE𝜖4 do not develop AD (Farrer et al., 1997). Other coding variants that increase susceptibility for AD include, but are not limited to Karch et al. (2014), proteins involved in innate immune responses such as the triggering receptor expressed on myeloid cells 2 (TREM2), PLCG2 and ABI3 (Sims et al., 2017) and variants of ABCA7 (Steinberg et al., 2015). Regardless of the lifestyles, or genetic predisposition associated with the onset of AD, the common manifestations and physiological symptoms involve inflammation and oxidative stress, the effectors of AD risk factors that will be further discussed in this review.

### Parkinson’s Disease

Parkinson’s Disease is the leading movement disorder and estimates suggest that more than 1 million people in the United States live with PD. More than 60,000 new PD cases are diagnosed each year; a prevalence expected to double in line with an aging population by 2040 (Kowal et al., 2013). Like AD, there is a substantial economic burden associated with PD. Estimated costs are over $25 billion per year for treatment, lost income and social security measures (Mayo Foundation for Medical Education and Research [MFMER], 2017). Approximately 10% of PD cases have a familial origin and can be traced to a small number of genetic mutations resulting in early-onset PD (Thomas and Beal, 2007). Leucine-rich repeat kinase 2 (LRRK2) is the most frequently mutated autosomal dominate gene sufficient for familial and some sporadic PD cases. Although the pathological mechanism of LRRK2 mutations has yet to be fully elucidated (Dachsel et al., 2010), LRRK2 regulates microglia activity and inflammatory responses (Kim et al., 2012). Genetic variations in β-glucocerebrosidase (GBA) and PARK-designated genes also notably increase the risk for development of PD-associated neuropathologies. PINK1 (PTEN-induced putative kinase 1) and its binding partner Parkin are also commonly mutated proteins in PD that normally maintain the integrity of the mitochondrial membrane and initiate autophagy of damaged mitochondria; however, when mutated, they fail to eliminate damaged mitochondria contributing to the accumulation of respiration-associated oxidants (Kondapalli et al., 2012). Based on epidemiological evidence, non-monogenic sporadic PD involves the exposure to pesticides neurotoxic to dopaminergic neurons (L’Episcopo et al., 2018). Exposure to such compounds elevates cellular oxidative potentials and mitochondrial dysfunction (McCormack et al., 2002), inflammation (Dzamko et al., 2015) and primes neuroglial cells toward activated phenotypes (Wu et al., 2002). Thus, mutations and neurotoxins implicated in PD can promote the accumulation of oxidative stress by compromising mitochondrial integrity, increasing ROS production or mediating inflammatory pathways. Such molecular signals promote canonical neuropathological signatures of PD including the aggregation of α-synuclein or activation of microglia activation in dopaminergic circuits. Therefore, life style factors that similarly increase oxidative stress and inflammation will aggravate PD disease progression.

### Chronic Traumatic Encephalopathy

Chronic Traumatic Encephalopathy (CTE) is a neurodegenerative disorder most prevalent in individuals who have experienced repetitive brain traumas or acute traumatic brain injury (TBI). Athletes who play contact intensive sports often resulting in concussions such as professional football players or soldiers exposed to military grade blasts are at an increased risk for developing CTE compared to the general population (Omalu, 2014). TBI does not always predispose individuals to CTE, but are highly correlative. Pathological examination of post-mortem brain tissue from athletes and military veterans exposed to blunt force trauma resulted in the diagnosis of CTE in 63% of the subjects, while 11% were diagnosed with AD (McKee et al., 2013). As suggested by this study, CTE can be comorbid with other prevalent neurodegenerative disorders like AD and fronto-temporal dementia, and often share neuropathologies and activated neuroinflammatory pathways. Instances of TBI are also prevalent in everyday life, as estimates found that in 2010 approximately 2.5 million people in the United States experienced a TBI that required a hospital visit (National Hospital Discharge Survey [NHDS], 2010) with estimations for the cost of acute care for TBI related injuries alone are over $60 billion annually in the United States (Hendricks et al., 2012). Thus, like other neurodegenerative conditions, risk factors for CTE exert significant health and economic burdens on society.

Patients diagnosed with CTE initially present with insidious symptoms associated with cognitive dysfunction including irritability, impulsivity, depression and short-term memory loss (McKee et al., 2009). As the disease progresses individuals develop dementia-like symptoms that parallel increased tauopathy signatures, multifocal axonal varicosities, and axonal loss. Similar genetic predispositions also appear between AD and CTE. Notably as for AD, the APOE𝜀4 allele confers a significant risk for developing CTE associated dementia when previously exposed to mild TBI (Sundström et al., 2007). An increased risk to develop CTE-like neuropathologies in APOE𝜀4 allelic populations was similarly observed in a cohort of boxers, who throughout their professional career had been exposed to significant head trauma (Jordan et al., 1997).

## Neurodegenerative Disorders and Their Pathological Markers

### Alzheimer’s Disease

Neuropathological surveys of patients with advanced AD observe pervasive neurodegeneration and distinct neuropathological features including intercellular neurofibrillary tangles composed of hyperphosphorylated tau and intracellular senile plaques composed of Aβ42 (Magalingam et al., 2018). Neurodegeneration in AD patients is initially selective for cholinergic neurons in the Nucleus Basalis of Meynert of the basal forebrain (Whitehouse et al., 1982; Arendt et al., 1985; Liu et al., 2015). Selective degeneration of cholinergic fibers in early phase AD is consistent with initial symptoms of memory loss and decreased executive functioning (Schmitz and Nathan Spreng, 2016) and cortically projecting axons from caudal part of this nucleus primarily innervate neocortical neurons in the temporal lobe, which also undergo neurodegeneration in early-phase AD. As AD progresses, neurodegeneration is evident by the thinning of the neocortical plate and the magnitude of thinning is proportional to AD symptomatology (Dickerson et al., 2009). Neurodegeneration in AD also involves synaptic depletion and retraction of axonal fibers, which can facilitate neuronal apoptosis (Lista and Hampel, 2017). Advanced AD is also pathologically characterized by increased permeability of the blood brain barrier (BBB), as confirmed by multiple post-mortem tissue analyses (Sweeney et al., 2018).

Elevated markers of inflammation and oxidative stress are pervasive in all brain regions in AD and serves as important biomarkers and potential pathological targets, as they promote Aβ deposition, microglia activation, and BBB permeability (Heneka et al., 2015). The oxidative stress biomarker 8-oxoguanine, a DNA lesion produced by hydroxy oxidation of guanine, exhibits abundant expression patterns in post-mortem AD tissue (Sheng et al., 2012). Additionally, aberrant regulation of cytosolic redox states in AD is evident by elevated oxidized lipid compounds such as 4-hydroxynonenal (Sayre et al., 2002), acrolein (Lovell et al., 2001) and 4-hydroxyhexenal (Bradley et al., 2012) in the hippocampus of AD patients. The magnitude of oxidative biomarker expression was a function of AD symptom severity, which supports the notion that inflammation and oxidative stress are neurotoxic self-reinforcing mechanisms that promote cytoarchitectural and morphological alterations observed in AD patients. Two of the cytokines notably upregulated in AD post-mortem tissue samples are IL-1β and IL-18 (Mrak and Griffin, 2001; Ojala et al., 2009). Their overexpression implicates activated inflammasome complexes in the generation of neuroinflammatory states, as both cytokines require proteolytic activation by inflammasome dependent caspase-1 activity from their zymogen forms; inflammasome complexes in AD will be described in more detail later. Of note, the brain is particularly susceptible to oxidative stress due to its high energy expenditure and oxygen consumption: estimations indicate that the brain uses up to 20% of the body’s circulating oxygen content (Chen and Zhong, 2014). Elevated inflammatory biomarkers from cerebrospinal fluid (CSF) and plasma of patients with mild-cognitive impairment (MCI), a prodromal condition of AD, indicate that in addition to end-stage AD, inflammation is also a preclinical condition of AD (Eikelenboom et al., 2002; Tarkowski et al., 2003), further validating its potential as a therapeutic target. As neuroinflammation also compromises the BBB in AD (Montagne et al., 2015), the resulting infiltration of peripheral monocytes into the brain, furthers the neuroinflammatory load in AD patients (Simard et al., 2006). The propagation of neuroinflammation in AD is also implicated as a driving force behind the aggregation of the pervasive Aβ and tau pathologies in AD brains (Simon et al., 2018).

Oxidative stress aggravates neuroinflammation via mechanisms involving microglial activation, which is an effector of aberrant synaptic pruning and synapse loss (Cai et al., 2014), a prominent AD neuropathology. Activation of microglia is in part triggered by Aβ protofibril deposits (Banerjee et al., 2011) and activation of microglia in AD has been confirmed by both *in vivo* imaging techniques (Garraux et al., 2013) and post-mortem tissue analysis (Solito and Sastre, 2012). The recruitment of peripheral monocytes into the brain by neuroinflammation, contributes to the production of sterile insult signals, such as HMGB1 (Rouhiainen et al., 2004). In addition to oxidative stress, sterile insult signals act as a second important facilitator of neuroinflammation in AD as HMGB1 upregulates production of inflammasome components, which when activated constitute a primary effector of microglia-mediated inflammation. Indeed, inappropriate activation of the NLRP3 inflammasome contributes to the pathogenesis of AD (Tan et al., 2013). The inflammasome also drives AD pathologies by recognizing and translating Aβ into inflammation; phagocytosis of Aβ by microglia stimulates lysosomal breakdown and consequent release of cathepsin B, an endogenous trigger of NLRP3 inflammasome activation that promotes neurotoxic IL-1β and caspase-1 release (Halle et al., 2008), which is essential for the downstream neuroinflammatory events observed in the AD brain. Indeed, there is a highly upregulated expression of caspase-1 in human MCI and AD brains and mice carrying Nlrp3(-/-) or Casp1(-/-) mutations were largely protected from the neural deficits associated with early-onset AD (Heneka et al., 2013). IL-1β has a particular association with AD because in both mouse models and human, it has been shown responsible for cognitive deficits, as measured by MMSE, (Tarkowski et al., 2003) and tau pathology in triple transgenic AD mice (Kitazawa et al., 2011). Additionally, IL-1β may also prime neurons to undergo excitotoxic death by the recruitment of MAPKs and potentiate glutamate-induced neurotoxicity (Tan et al., 2013).

### Parkinson’s Disease

PD is a multifactorial disease characterized by the progressive degeneration of dopaminergic neurons in the substantia nigra pars compacta leading to motor deficits including shaking, bradykinesia, rigidity, depression, dementia, and digestive difficulties (Jankovic, 2008). The pervasive effects and complexity of PD generates a battery of non-motor symptoms including olfactory deficits, sleep disturbances, depression, gastrointestinal disorders, and cognitive decline, all of which worsen as a function of disease severity (O’Sullivan et al., 2008). Aggregated α-synuclein fibrils are the predominate neuropathology associated with clinical manifestations of PD (McCann et al., 2014). It is important to recognize, however that a subset of patients with LRRK2 missense mutations exhibit nigral degeneration and PD symptomology without α-synuclein aggregates, commonly found in Lewy bodies (Giasson et al., 2006). α-Synuclein is a presynaptic protein capable of self-aggregating when mutated or misfolded due to certain point mutations or multiple repeats resulting in Lewy bodies and Lewy neurites (Olanow and Schapira, 2013). α-Synuclein accumulation begins early in PD development in the brain stem and then spreads to the dorsal motor nucleus of the vagus nerve, locus coeruleus to the nucleus accumbens in a caudal-rostral migration (Olanow and Schapira, 2013). Elevated α-synuclein inclusions in presynaptic regions leads to loss of cellular homeostasis and neuronal death by stimulating neurotoxicity, activation of inflammatory pathways and microglial activation (Jin et al., 2014). Indeed, patients with PD exhibit preferential activation of microglia in the substantia nigra region (McGeer et al., 1988), which correlates with the level of neuronal degeneration (Imamura et al., 2003). The α-synuclein aggregates also co-localize where there are higher numbers of activated microglia stimulating the conversion to a M2-like activated phenotype, known to potentiate inflammatory environments (Doorn et al., 2014). Interestingly, dopamine is an endogenous inhibitor of inflammasome activation (Yan et al., 2015) so the loss of dopamine characteristic of PD may drive inflammasome activation and consequently the elevation in inflammation and oxidative stress.

In line with the activation of microglia as a pathological signature of PD, activation of the inflammasome is a major source of neuroinflammation in PD. In both MPP+ and α-synuclein transgenic mouse PD models, elevated expression of NLRP3, caspase-1, and IL-1β have been observed in neurons while rotenone, a key pharmacological inducer of PD, induced strong upregulation of inflammasome activation *in vivo* in a manner dependent on Cdk5 (Zhang et al., 2016). Interestingly, α-synuclein, only when found as pathological aggregates, stimulates NLRP3 inflammasome activation via its interaction with TLR2 (Gustot et al., 2015). Another study attributed the microglial phagocytosis of α-synuclein as a trigger for NLRP3 inflammasome activation and consequent elevation of IL-1β levels (Codolo et al., 2013). Through another mechanism, MPTP-induced PD neuronal loss was exasperated by uncoupling protein 2 knockout, a protein controlling mitochondrial integrity and oxidative stress production, due to, in part, elevated inflammasome activation (Lu et al., 2014). Finally, in an *in vivo* PD model using both intracranial injection of 6-OHDA or LPS, inflammasome activation was found to be elevated at the sites of injection correlated with motor deficits in the rats. Interestingly, treatment with a caspase-1 inhibitor (Ac-YVAD-CMK) inhibited the motor deficits and improved neuronal survival to both the OHDA- and LPS-induced PD pathology indicating that inflammasome activation and its downstream pathways are critical determinates for PD pathologies (Mao et al., 2017). Based on this mounting evidence suggesting the role of the inflammasome in PD pathology, one group used rifampicin, a compound with neuroprotective properties, to show that pretreatment with rifampicin prevented rotenone-induced cytotoxicity in microglia by the prevention of NLRP3 activation and consequent caspase-1 activity (Liang et al., 2015). The final study indicates that therapeutic strategies that inhibit inflammasome activation have the potential to reduce PD-induced pathologies.

### Chronic Traumatic Encephalopathy

CTE can be distinguished by unique neuroanatomical features that are also consistent with heightened neuroinflammation and persistent oxidative stress. High-resolution T1 volumetric MRI scans have aided in the differentiation of CTE from other neurodegenerative disorders by identifying aggregated phosphorylated tau proteins, which is a unique neuropathological signature of CTE, as opposed to Aβ deposition together with tau in AD (Small et al., 2013). Further neuroimaging studies found that athletes who experienced head trauma have greater reductions in hippocampal volume (Singh et al., 2014; Bernick et al., 2015) as compared to age-matched non-football-player controls. Other studies have similarly demonstrated that TBI increases the risk to develop CTE associated neuropathologies (Gavett et al., 2010) and causes a reduction in the brain volume of the entorhinal cortex, the amygdala as well as less severe degrees of neuronal loss in the subcallosal and insular cortex, olfactory bulbs, mammillary bodies, locus coeruleus, substantia nigra, medial thalamus, and cerebral cortex. Notably, the pronounced involvement of subcortical and brainstem structures is unique to CTE among other neurodegenerative conditions. The neuropathological manifestations in confirmed CTE patients progress in proportion to clinicopathological features, such as memory loss and increased tendency for emotional lability, aggression, and violent outbursts (McKee et al., 2009). The clinical aspects of CTE match the apparent preferential neurodegeneration of brain regions associated with emotional processing and attention.

At the cellular level, patients with CTE exhibit an accumulation of phosphorylated tau protein as neurofibrillary tangles (NFTs), neurites, and glial tangles (GTs) in both cortical and subcortical structures. Tau deposition in CTE has distinguishable features from those in patients with AD (Schmidt et al., 2001). In CTE, the density of NFT and GT clusters is greater (Gavett et al., 2010), there is a distinct pattern of clustering of tau-immunoreactive abnormalities in brain perivascular regions, and the clustering is specific to superficial upper cortical layers responsible for corticocortical connections (McKee et al., 2009; Costanza et al., 2011). In addition to unique patterning of tauopathies in CTE, subjects with TBI-induced CTE have increased expression of the TAR DNA-Binding Protein 43 (TDP-43), which is prominent in the neurites and intranuclear regions of neurons and glia. A subset of CTE patients that have TDP-43 proteinopathy in spinal cord neurons are at increased risk for developing motor neuron degenerative conditions (McKee et al., 2010). Inflammation and reactive microglia are considered to significantly contribute mechanistically to CTE. In line with this, microglia activation was evident up to 17 years following a TBI (Ramlackhansingh et al., 2011) and implicates TBI as a priming event for persistent neuroinflammatory conditions and white matter degeneration that progresses as a function of time (Johnson et al., 2013). Patients with Dementia Pugilistica, a condition associated with repetitive TBI’s, also exhibit inflammatory pathologies such as heightened microglial activation and significant C1q labeling of neurons, along with substantial neurofibrillary tangles (NFTs). Inflammation may thus be a key inciting as well as propagating feature of DP neuropathology. As the severity of the clinical symptoms progresses through the course of CTE as the neurodegeneration increases (Stern et al., 2011), interventional treatments that target neuroinflammatory mechanisms may play an important role in preventing the progression of CTE-like neurodegenerative conditions.

### The Interplay of Oxidative Stress and Inflammation in Neurodegeneration

**FIGURE 1:**
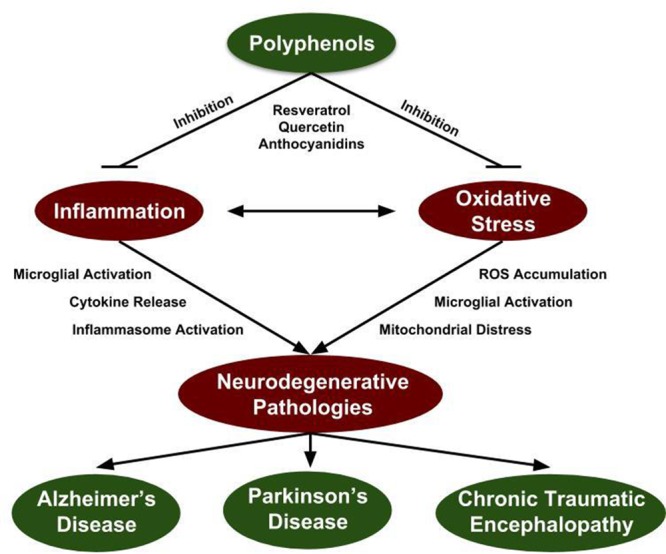
Polyphenol inhibition of neurodegenerative mechanisms. Inflammation and oxidative stress are prevalent within all neurodegenerative disorders and constitutes a potential target for therapeutic intervention. Since induction of inflammation and oxidative stress create a positive feedback loop, targeting both mechanisms is essential to quench their elevated state in the context of neurodegenerative disorders. Grape-derived polyphenol compounds have antioxidant and anti-inflammatory properties identifying them as a potential therapeutic approach for attenuation of neurodegenerative disorders.

Inflammation and oxidative stress are both age-related conditions that mutually aggravate the neuropathological features of AD, PD, and CTE. Notably, there is significant molecular crosstalk between the regulatory mechanisms of inflammation and oxidative stress creating a feedback loop of aggravating phenotypes (**Figure 1**). Inflamm-aging was a special term coined by Francheschi et al. which suggested that age-related chronic low-grade inflammation is asymptomatic, but overtime the effects compound to promote altered cellular function (Franceschi et al., 2006). Immunosenescence describes the aging of the immune system, which has reduced ability to respond to new antigens and mount an appropriate immune response and is considered one consequence of age-induced inflammation (Pawelec, 2017). This includes the inability to remove senescent cells as the reduced expression of TLRs and phagocytic activity of macrophages compromises their recognition by immunomodulators (Costantini et al., 2018). This is particularly important considering the crosstalk between inflammation and oxidative stress increases as a function of age, particularly from the failure of mitophagy, which leads to the accumulation of malfunctioning mitochondria that produce more ROS (Lionaki et al., 2015). ROS accumulation leads to damaged cells, consequent activation of the inflammatory mediators activated protein-1 and nuclear factor kappa B (NFκB) which eventually leads to a cellular senescent state (López-Otín et al., 2013). Although senescent cells are normally removed by the immune system, as a consequence of age-mediated immunosenescence and the reduced activity of immune cells, senescent cells accumulate, release proinflammatory cytokines and cyclically drive inflamm-aging (Fougère et al., 2017). This is particularly important considering that oxidative stress and inflammation are mutually aggravating one another increasing the risk for disease and serve as risk factors for neurodegeneration.

## Current Treatments for Neurodegeneration

### Alzheimer’s Disease

Current therapies for AD treat disease symptoms and final manifestations including Aβ and tau aggregates, memory loss, imbalances in acetylcholine levels, behavioral changes or sleep deficits. The prevailing therapeutic strategy for symptomatic dementia associated with AD involves acetylcholinesterase inhibitors (ChEi’s) that compensate for acetylcholine deficits and loss of projection cholinergic neurons in the basal forebrain. Toxicity associated with upward titration and long-term use limits their prolonged clinical utility, and over time AD neuropathologies progress in spite of treatments with ChEI’s (Hogan, 2014). From a different perspective, repurposing of existing therapeutic compounds with other primary indications for AD has not only provided insight into the mechanism of AD, but also exhibited initial clinical efficacy. One promising therapy involving direct intranasal insulin improved cognition (Reger et al., 2008) and reduced key markers of AD including amyloid, tau, and decreased glucose metabolism (Craft et al., 2012). Another strategy involves leveraging monoclonal antibodies targeting different Aβ species (i.e., monomer, oligomer, protofibril, and fibril) to recruit the host’s immune system to eliminate extracellular amyloid aggregates (Broadstock et al., 2014). In spite of their effectiveness in clearing amyloid plaques, they failed to improve cognitive function or impede neurodegeneration (Honig et al., 2018). The preliminary results from these trials indicate that plaque pathologies may be a consequence rather than an effector of neurodegenerative disorders. Other therapies in clinical trial aim to eliminate Aβ plaques by inhibiting the γ- or β-secretases responsible for producing them. These studies have either shown little success in the clinic (Fleisher et al., 2008; Coric et al., 2012) or demonstrate high levels of adverse side-effects such as in the case of the BACE1 inhibitor MK-8931 (Cai et al., 2012; Broadstock et al., 2014). Elimination of tau aggregates is another treatment paradigm and one compound, methylene blue, is in Phase III clinical trials to prevent the tau-tau binding.

As inflammation plays an important role in the etiology of AD pathologies, several anti-inflammatory therapies have been tested in clinical settings. For instance, long-term use of NSAIDs decreases AD development, slows disease progression, and improves cognitive functioning (Andersen et al., 1995; Laura et al., 2004). Likewise, ibuprofen and flurbiprofen potently ameliorate Aβ burden via modulation of γ-secretase and invoking anti-inflammatory effects possibly through the regulation of COX-2 (Carreras et al., 2013). In more robust clinical trials however, NSAIDs and other anti-inflammatory drugs exhibited limited clinical efficacy (Thal et al., 2005; Soininen et al., 2007) including the COX-2 inhibitors naproxen and celecoxib (Lyketsos et al., 2011) as well as ibuprofen (Pasqualetti et al., 2009). Azeliragon is a novel small molecule inhibitor against the Receptor for Advanced Glycation End products (RAGE): a receptor that initiates inflammatory cascades in response to sterile insults. Azeliragon is presently in phase 3 clinical trials toward slowing progression in patients with mild AD where preclinical trials have shown Azeliragon to decrease Aβ plaque deposition while promoting anti-inflammatory effects and reducing cognitive decline (Burstein et al., 2018).

Several antioxidant therapies have been tested in AD patients but with little success. Although supplementation with vitamin E and C in addition to cholinesterase inhibitors reduced levels of LPO in the blood, there were no changes in cognition (Arlt et al., 2012). Similarly, treatment with vitamin E, C, and a-lipoic acid reduced LPO levels though had no impact on Aβ42 or phosphorylated tau (Galasko et al., 2012). Interestingly, the polyphenolic compound curcumin, previously shown to inhibit Aβ aggregation and have antioxidant capabilities in AD animal models (Kenjiro et al., 2004), has shown no success in human clinical trials (Baum et al., 2008). Similarly, Ginkgo biloba, another polyphenolic-rich plant with antioxidant properties, showed no significant effect on cognitive decline in aging individuals (Snitz et al., 2009). The potential yet multiple failures to develop an effective AD therapy using antioxidants therefore indicates that the current strategies need revision, and emphasize the importance of targeting presymptomatic oxidative stress and inflammation. It is also possible that the antioxidant therapy may not cross the BBB where it is required to have an effect; likewise singularly reducing oxidative stress may not be sufficient to alleviate the symptoms of AD as neuroinflammation alone can increase oxidative load.

Therapeutic approaches under clinical development for AD target the neuroimmune system, microglia activation, or proteins in neuroinflammatory cascades. Mutations in the heterozygous triggering receptor expressed on myeloid cells (TREM2) represent an emerging genetic risk factor for AD (Pottier et al., 2013) and implicates microglia activity as contributing to AD development. The exact role TREM2 plays in innate immune responses is a matter of debate with evidence suggesting it is responsible for plaque uptake by microglia, but other studies found that TREM2 deficiency decreases neuroinflammation and protects against plaque induced neuronal toxicity (Leyns et al., 2017). An antibody targeting TREM2 activity is currently under preclinical development for AD. The receptor-interacting serine/threonine-protein kinase 1 (RIPK1) is another protein involved in microglia initiated neuroinflammation, is elevated in microglia of post-mortem cortical samples from AD patients (Ofengeim et al., 2017), and is being targeted by small molecular inhibitors in a Phase 1 clinical trial. Another anti-inflammatory approach for AD involves an inhibitor of NLRP3 inflammasome assembly, which in preclinical studies reduced Aβ plague deposition, memory impairment and microglia activation in an APP/PSEN1 murine model of AD. Initial preparations are underway to test this compound in a Phase I trial for AD.

### Parkinson’s Disease

The predominant symptomatic therapies for PD increase intrasynaptic concentrations of dopamine in degenerating dopamine nigrostriatal circuits. Dihydroxyphenylalanine (L-DOPA) is the molecular precursor of dopamine and its decarboxylation by tyrosine hydroxylase increases levels of intrasynaptic dopamine. Treatment of PD with formulated L-DOPA is the most common monotherapy for PD motor impairments (Poewe et al., 2010). The breakdown of dopamine by monoamine oxidase B (MAO-B) is another enzymatic pathway pharmacologically targeted for treatment of PD inhibiting the breakdown of intracellular dopamine. Inhibitors of MAO-B are often provided as adjunct therapy to L-DOPA supplementation to increase their efficacy (Moussa et al., 2017). Direct agonism of D1 dopamine receptors improves axial motor symptoms and dyskinesia in PD, but is limited in efficacy compared to MAO-B and L-DOPA treatment as independent preliminary randomized placebo-controlled studies involving dopamine receptor agonists pramipexole and ropinirole elicited no disease modifying effect in patients with early stage PD (Bonuccelli et al., 2009). Thus, treatment with dopamine surrogates does not prevent progression of underlying PD neuropathologies as increased dosing is required to maintain therapeutic efficacy (Poewe et al., 2010).

Alternative therapeutic approaches for treatment of PD focus on neuron and microglia metabolism and the reduction of synuclein plaques. Creatine monophosphate is an energy source during periods of cytosolic ATP depletion and has neuroprotective attributes in preclinical models of PD (Yang et al., 2009). However, in several clinical trials, creatine supplementation failed to improve PD associated symptoms (Mo et al., 2017). In addition to its antimicrobial activity, minocycline was shown to decrease microglia mediated neuroinflammation in models of PD, but a clinical trial failed to show improvements in PD symptomatology (Ninds Net-Pd Investigators, 2008). Immunotherapeutic approaches in PD target PD-specific extracellular protein aggregates, such as α-synuclein. Two antibodies, BIIB054 and PRX002, show preliminary efficacy in removing plasma α-synuclein in humans and clearing the protein aggregates from brain regions in mouse models of PD (Valera and Masliah, 2016; Brys et al., 2018) and both therapies are currently under Phase 2 development for PD.

Other novel therapeutic approaches under clinical development for PD target neuroinflammatory pathways. Gene polymorphisms of the LRRK2 protein are among the most prevalent causes of late-onset autosomal dominant and sporadic PD; the protein is highly expressed in macrophages, monocytes, and neutrophils and mutations result in hyperinflammatory responses from innate immune cells (Gillardon et al., 2012). The LRRK2 kinase is closely related to the receptor-interacting protein (RIP) kinases, which are key regulators of inflammasomes (Liu W. et al., 2017). Experiments with toll-like receptor 4 (TLR4)-stimulated rat primary microglia show that inflammation increases LRRK2 activity and expression, while inhibition of LRRK2 kinase activity or knockdown of protein expression attenuates TNFα secretion and iNOS induction. From this and similar studies, an inhibitor of the LRRK2 is currently under clinical development for PD in patients with LRRK2 polymorphisms.

## Grape-Derived Polyphenolic Compounds Attenuate Risk Factors of Neurodegeneration

Current treatment paradigms for neurodegenerative disorders are limited by their poor long-term efficacy and significant side-effects, creating an urgent need to develop preventative therapies that target common pre-symptomatic risk factors such as oxidative stress and inflammation. In addition to serving as effective first line treatments for cancer (Neuroimaging et al., 2010) and multiple sclerosis (Nurmikko et al., 2007), polyphenols may also serve as a prophylactic treatment for neurodegenerative disorders, as they potently and simultaneously target inflammatory and oxidative pathways. Grapes in particular are abundant in bioactive polyphenols, which include resveratrol, lycopene, quercetin, and flavan-3-ols, each of which possesses potent antioxidant and anti-inflammatory activities (Pezzuto, 2008). Several clinical trials have been conducted outlining the benefits of grape-derived compounds (**Table 1**); however, more work is required to define the specific bioactive components of these formulations and how they suppress the underlying disease physiologies. Our group has previously outlined the benefits of polyphenol-rich grape extracts toward the prevention of AD (Pasinetti et al., 2015); in the following section we will expand on the application of specific grape-derived polyphenols toward the prevention of oxidative stress and inflammation in the context of AD, PD, and CTE while introducing potential new mechanisms of action through attenuation of inflammasome activation.

**Table 1:** Polyphenols in clinical trial: a list of clinical trials that tested grape-derived polyphenol compounds in the context of neurodegenerative disorders or oxidative stress.

Compound	Disease indication	Status	Dosage	Primary and secondary endpoints	Results	Study type	Clinical trial	Reference
Grape powder	Mild cognitive decline	Complete	76 grams of grapes/30 days	Cognitive decline, sVOI,SPM,	Elevated metabolism in certain brain regions correlated with improvements in attention/working memory	DB, PC	n/a	(Lee et al., 2017)
Concord grape juice	Mild cognitive impairment	Complete	Not defined	Measures of cognition	Improvement in verbal learning and non-significant enhancement of verbal and spatial recall	R, C, DB	n/a	(Krikorian et al., 2010)
Anthocyanin (Medox)	Inflammation + dementia prevention	Recruiting	320 mg/day for 24 weeks	CogTrack battery, plasma IL-1, IL-2, IL-6, TNF-a, flow mediated dilation by MRI	TBD	R, I, PG	NCT03419039	N/A
Anthocyanin	Mild dementia	Complete	200 ml/day	Blood pressure, inflammatory markers (CRP and IL-6)	Improvements in verbal fluency, short-term memory and long-term memory and reduction of blood pressure	R	n/a	(Kent et al., 2017)
Anthocyanin	Oxidative stress	Complete	240 ml/day tart cherry juice	Plasma F(2)-isoprostane levels	Reduced oxidative stress in older adults	DB, PC, CO	n/a	(Traustadottir et al., 2009)
Resveratrol	Alzheimer’s disease	Completed	500 mg/day, upto 1 g/day for 13 weeks	ADCS-ADL, MMSE, Plasma Cytokines, CSF αβ40, Volumetric MRI	Prevented declined MMSE scores, change in AD, and CSF Aβ42 levels	R,I, PG, DB	NCT01504854	(Turner et al., 2015; Moussa et al., 2017)
Resveratrol	Mood and cognitive performance	Completed	75 mg, twice daily	Cognition, cerebrovascular responsiveness, wellbeing	Resveratrol and other vasoactive nutrients may enhance mood and cognitive and reduce risk of developing dementia in post-menopausal women	R, PC	n/a	(Evans et al., 2017)
Resveratrol	Mild cognitive impairment	Completed	200 mg/day for 24 weeks	Learning and memory, MRI hippocampal volume, RSFC	Top of Form Higher RSFC between RA HIPP and RA CTx; preservation of LA HIPP volume bottom of form	R,I, PG, DB	NCT01219244	N/A
Resveratrol	Smoking inflammation	Completed	500 mg/day for 30 days	Plasma CRP, Triglyceride, TAS, IL-6, TNF-α	Increased TAS, reduced plasma CRP	R, I, CA, DB	NCT01492114	(Albini et al., 2012)
CocoaVia	Inflammation + prevention of decreased cognition	Active	900 mg/day for 24 weeks	Cognitive performance CRP, IL-1β, BOLD Response via MRI	TBD	R, I, PG, DB	NCT03030053	N/A

*Some trials involved single agent treatment or grape based solutions. MMSE, mini-mental status examination; RSFC, resting state functional connectivity; TAS, total oxidation status; COA, cross over assignment; OL, open lab; CRP, C-reactive protein; DB, double blind; IL, interventional; PG, parallel group; R, randomized; ADCS-ADL, Alzheimer’s disease cooperative study-activity of daily living; Hipp, Hippocampal; RA, right anterior; CTx, cortex; MMP9, metalloproteinases; MDC, macrophage-derived chemokine; SIRT1, sirtuin-1; sVOI, standardized volumes of interest, SPM, statistical parametric mapping.*

### Polyphenolic Compounds Alleviate Alzheimer’s Disease Neuropathologies

Our group characterized the composition of a Grape Seed Polyphenol Extract (GSPE) containing 79.2 mg/g of proanthocyanidin dimers, 33.7 mg/g of catechin, 17 mg/g of epicatechin, and 22.3 mg/g of gallic acid (Ho et al., 2018). Using this extract, we observed multiple neuroprotective effects including the inhibition of Aβ fibril formation, oligomerization, and aggregation *in vitro* (Ono et al., 2008). These results were replicated in an *in vivo* transgenic Tg2576 AD mouse model in which GSPE treatment over 5 months inhibited high-molecular weight Aβ aggregates in the brain, which paralleled an increased in cognitive function as assessed with the Morris water maze test (Wang et al., 2008). In particular, we found that GSPE reduced the 56 kDa Aβ aggregate which is specifically related to memory dysfunction in rats. This study also found no differences in the activity of α-, β- or γ-secretases or insulin-degrading enzyme (IDE) indicating that the clearance of Aβ was not affected and the mechanism of GSPE may be a result of preventing Aβ’s initial aggregation. The microbiome was necessary to synthesize brain bioavailable phenolic acids derived from GSPE, which emphasizes how reciprocal interactions along the gut-brain axis contribute to disease progression (Pasinetti et al., 2015).

Apart from Aβ aggregation, we have shown how GSPE attenuates the formation of tauopathies, another pervasive clinical manifestation in AD dementias. Non-covalent interactions between GSPE-derived polyphenols and tau residues were believed to be the mechanism of action for the prevention of tau peptide aggregation (Ho et al., 2009). Indeed, we showed in a Drosophila eye model of tau production that GSPE administration prevented the eye-specific neurodegeneration associated with tau formation (Pfleger et al., 2010). In a transgenic NLRP3 mouse model, GSPE administered in the drinking water reduced the accumulation of tau pathology while protecting mice against motor deficits associated with the neuropathology (Santa-Maria et al., 2012). Finally, in the TMHT transgenic mouse model of AD tauopathy, GSPE attenuated tau aggregation in the mouse brain by inhibiting both the phosphorylation and assembly of tau peptides (Wang et al., 2010).

As previously outlined, inflammation and oxidative stress are highly integrated and contribute to neuropathological progression in AD. Many studies have moved forward to establish how grape-seed derived polyphenols simultaneously manage oxidative stress and inflammation in model systems of AD. Procyanidin B2 is a highly expressed proanthocyanidin in grape seeds and was shown to inhibit inflammasome activation in LPS-stimulated THP-1 macrophages through the transcriptional inhibition of COX2 and iNOS and reduced production of IL-6 and nitric oxide (Neus et al., 2014). Trimethyltin-induced memory dysfunction after 30 days in rats was prevented by rutin (0.75%) treatment particularly by reducing the number of reactive microglia and pro-inflammatory cytokines (Koda et al., 2009). Gallic acid, another major compound in grapes, attenuated Aβ plaque-induced neuroinflammation in cultured microglia by inhibiting NF-κB acetylation and transcription of inflammatory cytokines (Mi-Jeong et al., 2011). Gallic acid also modulated upstream activation of the Aβ peptide fibril *in vitro* (Liu et al., 2013). Resveratrol is a stilbene, abundant in grapes, which has been extensively studied in the context of AD neuropathologies. Resveratrol promotes the clearance of Aβ plaques via an intracellular mechanism involving proteasome degradation of Aβ (Marambaud et al., 2005). Resveratrol also has multiple antioxidative capacities including direct sequestration of free radicals, inhibition of nicotinamide adenine dinucleotide phosphate oxidase and myeloperoxidase gene transcription and promotion of the antioxidant enzymatic activity of SOD, catalase, glutathione and thioredoxin (Carrizzo et al., 2013). Resveratrol was also shown to inhibit neuroinflammation, which was demonstrated in *in vitro* LPS-mediated activation of astrocytes and microglia evidenced by reduced release of TNFα and nitric oxide (Bi et al., 2005). In the context of AD, neuroinflammation induced by treatment of Aβ1-42 in astrocytes and N9 microglial cell lines was dose-dependently inhibited by resveratrol treatment by regulating NF-κB translocation and p-IκB expression (Zhao et al., 2018). In D-galactose-mediated AD in rats, resveratrol (40 or 80 mg/kg body weight) inhibited Aβ1-42 fibril formation, neuroinflammation, accumulation of advanced glycation end-products (AGEs) and increased the integrity of the BBB (Claudin 5) indicating that *in vivo*, resveratrol can simultaneously alleviate neuroinflammation and oxidative stress in an AD model (Zhao et al., 2015). There are a handful of clinical trials using resveratrol in the context of AD that have attempted to validate their preclinical efficacy. The impact of daily resveratrol treatment with a dose increment of 500 to 2000 mg per day over 52 weeks in 119 patients with mild to moderate AD revealed that resveratrol penetrated the BBB in low micromolar amounts and attenuated the progressive decline of Aβ40 in the CSF, a factor associated with elevated activity of daily living (Sawda et al., 2017). In a follow-up study, resveratrol was shown to markedly reduce MMP9 CSF levels and increase macrophage-derived chemokine (MDC), IL-4, and fibroblast growth factor-2. Compared to baseline, resveratrol increased plasma MMP10, decreased IL-12 and RANTES indicating that the beneficial impact of resveratrol could be working through the suppression of neuroinflammation (Moussa et al., 2017). In contrast, another clinical trial that provided 500 mg of resveratrol to patients with mild to moderate AD neither decreased plasma or CSF levels of Aβ40, nor prevented brain volume loss as determined by volumetric MRI (Turner et al., 2015). This dichotomy of effects of resveratrol support the notion that AD is a complex multifaceted disease that requires several risk factors to be simultaneously targeted by a successful therapeutic. Further, therapies such as resveratrol that target the pre-symptomatic AD pathologies need to be administered early in the disease progression to prevent the accumulation of pathogenic markers.

Composite polyphenol solutions exhibit unique antioxidative properties in AD model systems, and results described below indicate synergism between bioactive components to contribute to their effects. For example, a grape seed pomace rich in flavonoids at a dose of 2% *w/w* dry powder inhibited age-related and chemically induced increases of lipid peroxidation and DNA damage in rats.(Rho and Kim, 2006) In a chronic cerebral hypoperfusion model representative of AD pathologies, oral administration of GSPE (95% purity, procyanidine 95%, polyphenol >85%, free phenol 15–25%) for 1 month rescued memory deficits by restoring cholinergic neuronal function and repressing oxidative damage in the hippocampus (Chen et al., 2017). A grape powder treatment, containing 3.5 mg of gallic acid equivalents per gram determined by the Folin-Ciocalteu method, incorporated into the diet at low-dose (3%) or high-dose (6%) over 66 weeks increased spatial learning and memory performance, lowered expression of Aβ fibrils and β-secretase in aging rats fed high-fructose-high-fat diet. This long-term study indicated that the cognitive decline and neurodegenerative predisposition in aging rats due to poor diet can be prevented with dietary intervention containing grape extract (Chou et al., 2016). Finally, in a single-prolonged stress (SPS) model, grape powder containing 57.2 mg/kg of catechins and 566 mg/kg of anthocyanins, prevented SPS-induced behavioral and memory impairment in rats by preventing increased plasma corticosterone levels, reversing anxiety and depression behavioral deficits, while maintaining BDNF levels in the amygdala of SPS rats (Solanki et al., 2015). A grape seed proanthocyanidin extract consisting of catechin, epicatechin and epicatechin gallate, enhanced cognition and spatial memory in APP/PS1 transgenic AD mice in addition to reducing Aβ fibrils and tau hyperphosphorylation. These data corroborated the *in vitro* studies in PC12 cells where the GSPE extract alleviated Aβ cytotoxicity, apoptosis and increased the mitochondrial membrane potential indicating that the reduced cytotoxicity could be due to the elevated oxidative protection (Lian et al., 2016).

### Attenuation of Parkinson’s Neuropathologies by Grape-Derived Polyphenol Compounds

The antioxidant and antiinflammatory action of grape-derived polyphenols also alleviates neuropathologies and symptomologies observed in PD model systems. The common 6-hydroxydopamine (6-OHDA) toxic model of PD induces a state of chronic inflammation, mitochondrial dysfunction, and oxidative stress that contributes to disease onset and progression (Jin et al., 2014). When administered to the right striatum by unilateral injection, 6-OHDA elicits a selective loss of dopaminergic neurons in the substantia nigra resulting in dopamine depletion in the striatum associated with the common PD pathologies (Deumens et al., 2002; Jin et al., 2008). Within the context of this model system, 4 weeks of 100 mg/kg Grape Seed Extract (GSE) treatment in rats demonstrated a beneficial effect on the direct and indirect striato-thalamo-cortical pathways in PD coupled with improved motor disorders (Sarkaki et al., 2012). In another study, an extract (Regrapex-R) prepared from whole grapes containing polyphenols (450 mg), anthocyanins (12 mg), and resveratrol (100 mg) exhibited dose-dependent scavenging effects on ROS, protective effects on the enzymatic activities of the mitochondrial electron transport chain and pyruvate dehydrogenase in the liver mitochondria which protected transgenic α-synuclein expressing Drosophila PD models fed 0.08 mg/100 g culture media from loss of motor function (Long et al., 2009). Also, extracts prepared from grape seeds rich in anthocyanins and proanthocyanidins (total 820 mg/g polyphenols) were shown to protect rotenone-induced PD pathologies in a primary midbrain cultures by inhibiting dysfunctions in mitochondrial respiration (Strathearn et al., 2014).

Treatment with single grape-seed derived polyphenols similarly improved multiple neuropathological signatures in PD model systems. One study showed that each chrysin, catechin, gen strain, quercetin, and naringenin, in primary rat mesencephalic cultures, protected against oxidative stress-induced cell death via a number of stressors including hydrogen peroxide, rotenone, and the PD-associated neurotoxic *N*-methyl-4-phenyl-1,2,3,6-tetrahydropyridinium hydrochloride (MPP)+ (Mercer et al., 2005). Flavonoids in particular exhibited antioxidative characteristics, as the grape-derived flavonoids quercetin and catechin significantly reduced the onset of neuronal death induced by the exposure to ROS (Mercer et al., 2005). Myricetin, an abundant compound in grape extracts, also was shown to prevent α-synuclein oligomerization *in vitro* and prevent the subsequent synaptic toxicity in mouse hippocampal slices (Takahashi et al., 2015). Additionally, a 95% proanthocyanidins mixture containing 60–80% oligomers derived from grape seeds when administered to mice for 7 days effectively elevated serotonin dopamine and noradrenaline levels in the frontal cortex, hippocampus and hypothalamus, likely by inhibiting monoamine oxidase-A activity (Xu et al., 2010).

One study utilizing the 6-OHDA model in Sprague-Dawley rats showed that resveratrol treatment at 10, 20, and 40 mg/kg over 10 weeks preserved the motor deficits associated with dopaminergic neuron loss. These observations were also associated with a suppression of COX-2 activity and TNFα mRNA in the substantia nigra suggesting that resveratrol treatment attenuates elevated neuroinflammation in the PD brain (Jin et al., 2008). Resveratrol treatment at 20 mg/kg for three weeks presented significant protective effects in *in vitro* and *in vivo* PD models. In rats pre-treated for 15 days with resveratrol prior to a unilateral intrastriatal injection with 6-OHDA, there was a significant decrease in the levels of thiobarbituric acid reactive substances (TBARS), lipid peroxidation, and decreased activity of phospholipase A2. The investigators also reported a significant increase in expression and activities of antioxidants such as glutathione in the striatum (Khan et al., 2010). The authors speculated that by enhancing the presence of these antioxidants either directly or indirectly, resveratrol helped preserve dopaminergic neurons’ integrity by preventing dopamine autoxidation (Khan et al., 2010), a key mechanism of increased oxidative stress in dopaminergic neurons.

An important consideration toward neuroinflammatory mechanisms in PD, like AD, is the attenuation of microglia activation (Liu et al., 2003; Bureau et al., 2008). In an *in vitro* co-culture system of neuronal PC12 cells and N9 microglia activated by exposure to the endotoxin lipopolysaccharide (LPS), resveratrol and quercetin prevented apoptosis of the PC12 neurons in a manner dependent on the inhibition of microglial activation and consequent reduction of IL-1β and TNFα release (Bureau et al., 2008). Further, in rat primary midbrain neuron-glia cultures, resveratrol dose-dependently protected neuronal integrity to LPS-induced inflammation by preventing microglia activation and inhibiting NADPH oxidase, intracellular MAPKs and NF-κB signaling pathways that together promote heightened inflammatory responses (Zhang et al., 2010).

### Polyphenol Compounds in Chronic Traumatic Encephalopathy

The preclinical application of grape-derived polyphenol compounds for the treatment of CTE has produced mixed results likely due to the lack of samples and heterogeneity of the disease. Some successful studies include treatment of rats exposed to a moderate force fluid percussion injury model of TBI with the polyphenol quercetin. Treatment with quercetin after the injury maintained levels of glutathione, prevented the induction of the oxidative mediator myeloperoxidase, and preserved axonal integrity and function (Schültke et al., 2005). Pretreatment of mice with ellagic acid prior to a TBI in rats provided resilience against multiple TBI induced neuropathologies, including CTE. The administration of ellagic acid prevented memory and hippocampal long term potentiation impairments, and decreased the brain concentration of IL-1β and IL-6 (Farbood et al., 2015). Whether or not ellagic acid maintained BBB integrity by reducing brain inflammatory cytokines is an important line of future investigation that can provide insight to TBI neuropathologies. Acute treatment with resveratrol following TBI, in contrast to pretreatment, similarly prevented TBI-induced activation of microglia in the cerebral cortex, corpus callosum and hippocampus of rats, while also preventing TBI-mediated upregulation of inflammatory cytokines IL-6 and IL-12 in the hippocampus (Gatson et al., 2013). Oxyresveratrol, a compound structurally similar to resveratrol, also prevented TBI-induced neuropathologies (Andrabi et al., 2004). Experimental evidence indicates that grape-derived polyphenol compounds have the potential to provide relief for TBI-induced neuropathologies thereby decreasing the risk of developing CTE.

### Polyphenol Compounds as Free Radical Scavengers

**FIGURE 2:**
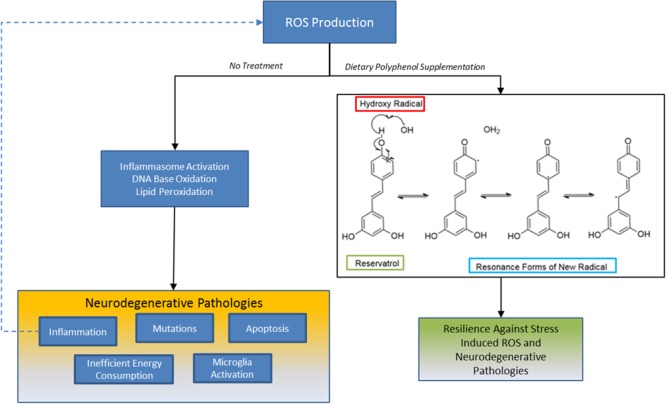
Sequestration of ROS by polyphenols: one mechanism grape derived polyphenols may benefit patients with presymptomatic neurodegenerative disorders are by buffering the redox state in neurons, microglia or astrocytes. Oxidative stress is a prominent pathology across all neurodegenerative disorders and its production by different stressors can result in self propagating loop of inflammation, DNA damage, and apoptosis. Polyphenols like trans-resveratrol inhibit ROS propagation by hydroxy radicals, for example, because its unique chemical structure affords the generation of energetically stable resonance radicals. This mechanism may partially contribute to neuroresilient traits of polyphenols.

Polyphenol compounds have inherent antioxidant characteristics due to the electron structure of their phenolic rings. Reactive oxygen species (ROS) can be derived from the mitochondria, as seen following lysosomal rupture, osmotic disequilibrium, or metabolic stress (Perron and Brumaghim, 2009), or can be of a non-mitochondrial origin, such as following the reaction of ionizing radiation with cellular components, metal oxidizers, and from NADPH oxidases or xanthine oxidase (Droge, 2002). The steady state of ROS in a cell is derived from mitochondria and shifting of this equilibrium toward the generation of more free radicals promotes the production of inflammatory cytokines by activating inflammasome complexes (Zhou et al., 2011). Polyphenol compounds can prevent this sequence of events through its inherent ability to sequester excessive ROS. Abundant ROS generated in the cytoplasm include superoxide anion radicals (^.^O_2_), hydrogen peroxide (H_2_O_2_), hydroxyl radical (^.^OH), alkoxyl radicals (^.^OR), or peroxyl radicals (^.^OOR). In addition to activating inflammasomes, ROS readily react with cellular lipids, carbohydrates, proteins and nucleic acids to produce toxic secondary species. Polyphenol compounds are strong antioxidants due to their characteristic aromatic ring structure that always contains at least one substituent hydroxyl group. Polyphenols eliminate strong ROS such as hydroxyl radicals through homolytic cleavage of the hydroxyl group, which delocalizes the singlet electron into the π orbitals of phenol ring and forms energetically stable resonance derivatives. The relatively higher reduction potential of the new radical compounds prevent further propagation of new radicals. Polyphenol compounds are diverse in the number of aromatic rings and their conjugating moieties, which increase or decrease their antioxidative properties, respectively. For example, a greater number of para- and ortho- substituent electron donating groups on aromatic rings decrease the hydroxyl dissociation energy, while more conjugated π orbitals in the planar structure increase resonance effects (Foti, 2007). Abundant grape derived polyphenolic compounds including resveratrol, quercetin, catechin and epicatechin, each would have strong antioxidative properties due to their conjugated π orbitals and increased number of hydroxyl groups (**Figure 2**). On a relative basis, it was previously reported that between the four polyphenol compounds mentioned, quercetin most readily sequesters the synthetic radical DPPH and the physiological peroxynitrate anion from solution followed by catechin, epicatechin and resveratrol (Rice-evans et al., 1995). Sequestration of free radicals by these polyphenols was amplified when administered synergistically. The superior free radical scavenging properties of quercetin relative to epicatechin and catechin may be attributed to additional resonance stabilization of the ketone-enol conjugated system. The unique structural properties of polyphenols may, therefore, provide them their strong anti-inflammatory effects and may act as a preventative treatment for neurodegenerative disorders that are propagated by ROS production and heightened inflammation.

### Polyphenols Inhibit Microglia Activation and Inflammasome-Mediated Inflammation

As indicated throughout this discussion, inflammasome-mediated neuroinflammation may contribute to overt neuropathological signatures in neurodegenerative disorders. Inflammasomes are a class of multimeric protein complexes that are responsible for initiating and propagating inflammation in response to endogenous or exogenous insult signals, such as alterations to intracellular redox potentials. The NLRP3 inflammasome is of particular interest for neurodegenerative disorders because experimental evidence indicates that it gates neuroinflammatory responses through its unique kinetic properties, which is different from other inflammasomes (Sutterwala et al., 2014). As neurodegenerative disorders are collectively characterized by progressive accumulation of neuroinflammatory markers in response to oxidative stress, dysregulated NLRP3 activity may represent a principal source of their neuroinflammatory pathologies.

Activation of the NLRP3 inflammasome requires a two-step paradigm whereby an initial stress signal promotes the transcription of the *Nlrp3* gene, while a second activation signal promotes assembly of the multimeric complex and its activation (Herman and Pasinetti, 2018). Upon activation, the caspase-1 domain of the NLRP3 inflammasome cleaves zymogen forms of IL-1β and IL-18 into their functional forms (Garlanda et al., 2013). Importantly, molecular intermediates downstream of oxidative and metabolic stress facilitate the assembly of the NLRP3 inflammasome. For example, ROS production, a characteristic of neurodegenerative pathology, is a potent signal for NLRP3 inflammasome assembly (Baughman et al., 2011; Chen et al., 2015). Cholesterol crystals and Aβ plaques, prominent in AD neuropathologies, similarly activate the NLRP3 inflammasome via the production of ROS (Rajamaki et al., 2010). Inhibition of NLRP3 activation by removing intracellular ROS through ROS scavengers represent a mean to mitigate presymptomatic systemic and neuroinflammation observed across neurodegenerative disorders. Specifically, the antioxidant properties of polyphenols outlined in *Section Polyphenol Compounds as Free Radical Scavengers* identify them as potential inhibitors of ROS mediated NLRP3 activation. Several studies provide evidence that grape-derived polyphenols can suppress inflammasome activity and the production of deleterious inflammatory cytokines (Hein et al., 2010). There is no evidence yet to indicate that polyphenolic compounds directly inhibit the NLRP3 inflammasome either through allosteric regulation of the complex or inhibition of complex assembly. Rather, evidence suggests that the sequestration of free radicals by polyphenols plays a critical role in the preventing the activation of the NLRP3 inflammasome.

In a subarachnoid hemorrhage model of TBI, the administration of resveratrol prevented cerebral inflammation and cortical apoptosis, events that were concomitant with reduced NLRP3 activity, microglia activation, and neutrophil infiltration into the brain (Zhang et al., 2017). Inhibition of the NLRP3 inflammasome and neuronal apoptosis via resveratrol in this model was mediated in part through sequestration of superoxide anion levels. Another study showed that resveratrol inhibited the accumulation of acetylated α-tubulin, which is a function of both cytosolic redox states and damage incurred by mitochondrial damage, thus preventing the proper assembly of the inflammasome components to elicit an inflammatory response (Misawa et al., 2015). Alternatively, the suppression of NLRP3 cytokine production and ROS by resveratrol in a separate rat TBI model was shown to be sirtuin 1 (SIRT1) dependent, illustrating the pleiotropic manner by which polyphenols suppress NLRP3 mediated neuroinflammation (Zou et al., 2018).

Quercetin inhibited the induction of inflammation by the toxin *E. coli* O157:H7 in epithelial cells by maintaining both mitochondrial integrity and by preventing the accumulation of mitochondrial ROS (Xue et al., 2017). In the monosodium urate model of gout arthritis, quercetin pre-treatment inhibited the inflammatory phenotype by, at least in part, inhibition of the NLRP3 inflammasome (Ruiz-Miyazawa et al., 2017). Catechin also inhibited MSU-induced IL-1β secretion and NLRP3 inflammasome activation in MSU-challenged THP-1 cells in a ROS-dependent manner (Jhang et al., 2015). Similarly, in a collagen-induced model of arthritis, quercetin inhibited inflammasome activation and associated inflammatory induction (Yang et al., 2018). The administration of the polyphenol quercetin prior to spinal cord injury in mice, which similarly decreased NLRP3 mediated cytokine production through the downregulation of ROS (Jiang et al., 2016). In a rat model of diabetes, increased intracellular ROS upregulated the thioredoxin-interacting protein (TXNIP), which facilitates activation of the NLRP3 inflammasome. Administration of quercetin to streptozocin-induced diabetic mice prevented TXNIP upregulation and inflammatory responses, highlighting the antioxidant characteristics of quercetin (Wang et al., 2013).

The procyanidins also possess anti-inflammatory activity, as illustrated through by their ability to inhibit inflammasome activation and reduce ROS formation in a LPS-induced model of human endothelial cell inflammation (Yang et al., 2014). Similar observations were evident following LPS-stimulation of macrophages (Neus et al., 2014). Like quercetin, procyanidins inhibited monosodium urate induced inflammation in mice by blocking inflammasome activation, IL-1β release and ROS production (Liu H.J. et al., 2017). In an attempt to develop a therapy against morphine-induced tolerance, procyanidins were found to increase sensitivity to morphine by inhibiting inflammasome activation in the spinal cord microglia (Cai et al., 2016). The shared ability of quercetin and procyanidins to prevent inflammasome activation through a reduction in ROS despite their distinct three-dimensional conformation and different conjugating moieties support a mechanism of action involving radical sequestration rather than protein-ligand interactions. Altogether, there is substantial evidence suggesting that the polyphenolic components of grape-derived extracts are potent inhibitors of the inflammasome, an important factor in neurodegenerative pathogenesis; however, further investigations are necessary to determine how grape-derived polyphenols can influence inflammasome activity in the context of neurodegenerative disorders.

Activation of inflammasomes parallels microglia reactivity. Studies were therefore conducted to determine if polyphenols simultaneously target NLRP3 inflammasome activation and microglia reactivity. One study found that intraperitoneal administration of dihydromyricetin attenuated the number of activated microglia in the hippocampus and cortex of APP/PS1 mice while reducing expression of NLRP3 inflammasome components and stimulating clearance of Aβ (Feng et al., 2018). Dual down regulation of these inflammatory pathways by the grape derived flavonoid dihydromyricetin also improved standard cognitive deficits observed in the APP/PS1 transgenic model of AD. Further studies should continue to characterize suppression of different innate immune pathways by polyphenols.

## Conclusion

While current therapies for Alzheimer’s disease, Parkinson’s disease and CTE provide acute symptomatic relief, neurodegeneration proceeds exponentially in the brain. Both oxidative stress and neuroinflammation precede overt symptomology of neurodegenerative disorders and each promotes neurotoxic environments associated with neurodegeneration. Dietary botanicals, in particular grape-derived polyphenols, have the potential to serve as an alternative treatment mode for the prevention of neurodegenerative disorders due to their ability to limit activation of oxidative pathways and inflammation following the exposure to stochastic environmental stressors. As reviewed, individual polyphenols have been shown both *in vitro* and *in vivo* to have potent antioxidant and anti-inflammatory activity that improve neuropathological features and behavioral phenotypes in neurodegenerative diseases. In particular, a previously untapped mechanism of neuroinflammation involving inflammasome activation may be a potential pharmaceutical target of polyphenolic anti-neuroinflammatory effects in the context of neurodegeneration. The studies to date are encouraging as they support the development of an optimized grape-derived polyphenol therapeutic that through its anti-inflammatory and antioxidant activities, may delay the onset or slow the progression of neurodegenerative diseases.

## Author Contributions

FH and SW contributed equally to the preparation and writing of the manuscript. JB assisted in the writing and composing of the manuscript. GMP reviewed and edited the contents of the manuscript.

## Disclaimer

The authors acknowledge that the contents of this study do not represent the views of the NCCIH, the ODS, the NIH, the U.S. Department of Veterans Affairs, or the United States Government.

## Conflict of Interest Statement

The authors declare that the research was conducted in the absence of any commercial or financial relationships that could be construed as a potential conflict of interest.

## References

[B1] Albini A. Tosetti F. Li V. W. Noonan D. M. Li W. W. (2012). Cancer prevention by targeting angiogenesis. *Nat. Rev. Clin. Oncol.* 9 498–509. 10.1038/nrclinonc.2012.120 22850752

[B2] Al-Chalabi A. van den Berg L. H. Veldink J. (2016). Gene discovery in amyotrophic lateral sclerosis: implications for clinical management. *Nat. Rev. Neurol.* 13 96–104. 10.1038/nrneurol.2016.182 27982040

[B3] Alzheimer’s Association (2016). 2016 Alzheimer’s disease facts and figures. *Alzheimers Dement.* 12 459–509. 10.1016/j.jalz.2016.03.001 27570871

[B4] Andersen K. Launer L. J. Ott A. Hoes A. W. Breteler M. M. B. Hofman A. (1995). Do nonsteroidal anti-inflammatory drugs decrease the risk for Alzheimer’s disease? *Neurology* 45 1441–1445. 7644037 10.1212/wnl.45.8.1441

[B5] Andrabi S. A. Spina M. G. Lorenz P. Ebmeyer U. Wolf G. Horn T. F. W. (2004). Oxyresveratrol (trans-2,3’,4,5’-tetrahydroxystilbene) is neuroprotective and inhibits the apoptotic cell death in transient cerebral ischemia. *Brain Res.* 1017 98–107. 10.1016/j.brainres.2004.05.038 15261105

[B6] Arendt T. Bigl V. Tennstedt A. Arendt A. (1985). Neuronal loss in different parts of the nucleus basalis is related to neuritic plaque formation in cortical target areas in Alzheimer’s disease. *Neuroscience* 14 1–14. 10.1016/0306-4522(85)90160-5 3974875

[B7] Arlt S. Müller-Thomsen T. Beisiegel U. Kontush A. (2012). Effect of one-year Vitamin C- and E-Supplementation on cerebrospinal fluid oxidation parameters and clinical course in Alzheimer’s Disease. *Neurochem. Res.* 37 2706–2714. 10.1007/s11064-012-0860-8 22878647

[B8] Banerjee S. Hellier J. Dewey M. Romeo R. Ballard C. Baldwin R. (2011). Sertraline or mirtazapine for depression in dementia (HTA-SADD): a randomised, multicentre, double-blind, placebo-controlled trial. *Lancet* 378 403–411. 10.1016/S0140-6736(11)60830-1 21764118

[B9] Baughman J. M. Perocchi F. Girgis H. S. Plovanich M. Belcher-Timme C. A. Sancak Y. (2011). Integrative genomics identifies MCU as an essential component of the mitochondrial calcium uniporter. *Nature* 476 341–345. 10.1038/nature10234 21685886 PMC3486726

[B10] Baum L. Lam C. W. K. Cheung S. K.-K. Kwok T. Lui V. Tsoh J. (2008). Six-Month randomized, placebo-controlled, double-blind, pilot clinical trial of curcumin in patients with Alzheimer Disease. *J. Clin. Psychopharmacol.* 28 110–113. 18204357 10.1097/jcp.0b013e318160862c

[B11] Benedict C. Byberg L. Cedernaes J. Hogenkamp P. S. Giedratis V. Kilander L. (2015). Self-reported sleep disturbance is associated with Alzheimer’s disease risk in men. *Alzheimers Dement.* 11 1090–1097. 10.1016/j.jalz.2014.08.104 25438949

[B12] Bernick C. Banks S. J. Shin W. Obuchowski N. Butler S. Noback M. (2015). Repeated head trauma is associated with smaller thalamic volumes and slower processing speed: the Professional Fighters’ Brain Health Study. *Br. J. Sports Med.* 49 1007–1011. 10.1136/bjsports-2014-093877 25633832 PMC4518758

[B13] Bi X. L. Yang J. Y. Dong Y. X. Wang J. M. Cui Y. H. Ikeshima T. (2005). Resveratrol inhibits nitric oxide and TNF-alpha production by lipopolysaccharide-activated microglia. *Int. Immunopharmacol.* 5 185–193. 10.1016/j.intimp.2004.08.008 15589480

[B14] Bonuccelli U. Del Dotto P. Rascol O. Calne D. Teychenne P. Leigh P. (2009). Role of dopamine receptor agonists in the treatment of early Parkinson’s disease. *Parkinson. Relat. Disord.* 15(Suppl. 4) S44–S53. 10.1016/S1353-8020(09)70835-1 20123557

[B15] Bradley M. A. Xiong-Fister S. Markesbery W. R. Lovell M. A. (2012). Elevated 4-hydroxyhexenal in Alzheimer’s disease (AD) progression. *Neurobiol. Aging* 33 1034–1044. 10.1016/j.neurobiolaging.2010.08.016 20965613 PMC3025307

[B16] Broadstock M. Ballard C. Corbett A. (2014). Latest treatment options for Alzheimer’s disease, Parkinson’s disease dementia and dementia with Lewy bodies. *Expert Opin. Pharmacother.* 15 1797–1810. 10.1517/14656566.2014.936848 24992196

[B17] Brys M. Ellenbogen A. Fanning L. Penner N. Yang M. Welch M. (2018). Randomized, double-blind, placebo-controlled, single ascending dose study of Anti-Alpha-Synuclein Antibody BIIB054 in Patients with Parkinson’s Disease (S26.001). *Neurology* 90.

[B18] Bureau G. Longpré F. Martinoli M. G. (2008). Resveratrol and quercetin, two natural polyphenols, reduce apoptotic neuronal cell death induced by neuroinflammation. *J. Neurosci. Res.* 86 403–410. 10.1002/jnr.21503 17929310

[B19] Burstein A. H. Sabbagh M. Andrews R. Valcarce C. Dunn I. Altstiel L. (2018). Development of Azeliragon, an oral small molecule antagonist of the receptor for advanced glycation Endproducts, for the potential slowing of loss of cognition in mild Alzheimer’s Disease. *J. Prev. Alzheimers Dis.* 5 149–154. 10.14283/jpad.2018.18 29616709 PMC12280790

[B20] Cai J. Qi X. Kociok N. Skosyrski S. Emilio A. Ruan Q. (2012). β-Secretase (BACE1) inhibition causes retinal pathology by vascular dysregulation and accumulation of age pigment. *EMBO Mol. Med.* 4 980–991. 10.1002/emmm.201101084 22903875 PMC3491829

[B21] Cai Y. Kong H. Pan Y. B. Jiang L. Pan X. X. Hu L. (2016). Procyanidins alleviates morphine tolerance by inhibiting activation of NLRP3 inflammasome in microglia. *J. Neuroinflammation* 13 1458–1470. 10.1186/s12974-016-0520-z 26931361 PMC4774188

[B22] Cai Z. Hussain M. D. Yan L.-J. (2014). Microglia, neuroinflammation, and beta-amyloid protein in Alzheimer’s disease. *Int. J. Neurosci.* 124 307–321. 10.3109/00207454.2013.833510 23930978

[B23] Carreras I. McKee A. C. Choi J. K. Aytan N. Kowall N. W. Jenkins B. G. (2013). R-flurbiprofen improves tau, but not Aß pathology in a triple transgenic model of Alzheimer’s disease. *Brain Res.* 1541 115–127. 10.1016/j.brainres.2013.10.025 24161403 PMC3863610

[B24] Carrizzo A. Forte M. Damato A. Trimarco V. Salzano F. Bartolo M. (2013). Antioxidant effects of resveratrol in cardiovascular, cerebral and metabolic diseases. *Food Chem. Toxicol.* 61 215–226. 10.1016/j.fct.2013.07.021 23872128

[B25] Cermakova P. Johnell K. Fastbom J. Garcia-Ptacek S. Lund L. H. Winblad B. (2015). Cardiovascular diseases in 30,000 patients in the Swedish dementia registry. *J. Alzheimers Dis.* 48 949–958. 10.3233/JAD-150499 26402118

[B26] Chen C. Zheng Y. Wu T. Wu C. Cheng X. (2017). Oral administration of grape seed polyphenol extract restores memory deficits in chronic cerebral hypoperfusion rats. *Behav. Pharmacol.* 28 207–213. 10.1097/FBP.0000000000000276 27984208

[B27] Chen L. Na R. Boldt E. Ran Q. (2015). NLRP3 inflammasome activation by mitochondrial reactive oxygen species plays a key role in long-term cognitive impairment induced by paraquat exposure. *Neurobiol. Aging* 36 2533–2543. 10.1016/j.neurobiolaging.2015.05.018 26119225

[B28] Chen Z. Zhong C. (2014). Oxidative stress in Alzheimer’s disease. *Neurosci. Bull.* 30 271–281. 10.1007/s12264-013-1423-y 24664866 PMC5562667

[B29] Chou L.-M. Lin C.-I. Chen Y.-H. Liao H. Lin S.-H. (2016). A diet containing grape powder ameliorates the cognitive decline in aged rats with a long-term high-fructose-high-fat dietary pattern. *J. Nutr. Biochem.* 34 52–60. 10.1016/j.jnutbio.2016.04.006 27206221

[B30] Codolo G. Plotegher N. Pozzobon T. Brucale M. Tessari I. Bubacco L. (2013). Triggering of inflammasome by aggregated α–Synuclein, an inflammatory response in synucleinopathies. *PLoS One* 8:e55375. 10.1371/journal.pone.0055375 23383169 PMC3561263

[B31] Coric V. van Dyck C. H. Salloway S. (2012). Safety and tolerability of the γ-secretase inhibitor avagacestat in a phase 2 study of mild to moderate Alzheimer disease. *Arch. Neurol.* 69 1430–1440. 22892585 10.1001/archneurol.2012.2194

[B32] Costantini E. D’Angelo C. Reale M. (2018). The role of immunosenescence in neurodegenerative diseases. *Mediat. Inflamm.* 2018:6039171. 10.1155/2018/6039171 29706800 PMC5863336

[B33] Costanza A. Weber K. Gandy S. Bouras C. Hof P. R. Giannakopoulos P. (2011). Review: contact sport-related chronic traumatic encephalopathy in the elderly: clinical expression and structural substrates. *Neuropathol. Appl. Neurobiol.* 37 570–584. 10.1111/j.1365-2990.2011.01186.x 21696410 PMC3166385

[B34] Craft S. Baker L. D. Montine T. J. Minoshima S. (2012). Intranasal insulin therapy for Alzheimer disease and amnestic mild cognitive impairment: a pilot clinical trial. *Arch. Neurol.* 69 29–38. 10.1001/archneurol.2011.233 21911655 PMC3260944

[B35] Dachsel J. C. Nishioka K. Vilarino-Guell C. Lincoln S. J. Soto-Ortolaza A. I. Kachergus J. (2010). Heterodimerization of Lrrk1-Lrrk2: implications for LRRK2-associated Parkinson disease. *Mech. Ageing Dev.* 131 210–214. 10.1016/j.mad.2010.01.009 20144646 PMC2847049

[B36] Deumens R. Blokland A. Prickaerts J. (2002). Modeling Parkinson’s disease in rats: an evaluation of 6-OHDA lesions of the nigrostriatal pathway. *Exp. Neurol.* 175 303–317. 10.1006/exnr.2002.7891 12061862

[B37] Dickerson B. C. Bakkour A. Salat D. H. Feczko E. Pacheco J. Greve D. N. (2009). The cortical signature of Alzheimer’s disease: regionally specific cortical thinning relates to symptom severity in very mild to mild AD dementia and is detectable in asymptomatic amyloid-positive individuals. *Cereb. Cortex* 19 497–510. 10.1093/cercor/bhn113 18632739 PMC2638813

[B38] Doorn K. J. Moors T. Drukarch B. van de Berg W. D. J. Lucassen P. J. van Dam A.-M. (2014). Microglial phenotypes and toll-like receptor 2 in the substantia nigra and hippocampus of incidental Lewy body disease cases and Parkinson’s disease patients. *Acta Neuropathol. Commun.* 2:90. 10.1186/s40478-014-0090-1 25099483 PMC4224021

[B39] Droge W. (2002). Free radicals in the physiological control of cell function. *Physiol. Rev.* 82 47–95. 10.1152/physrev.00018.2001 11773609

[B40] Dzamko N. Geczy C. L. Halliday G. M. (2015). Inflammation is genetically implicated in Parkinson’s disease. *Neuroscience* 302 89–102. 10.1016/j.neuroscience.2014.10.028 25450953

[B41] Eikelenboom P. Bate C. Van Gool W. A. Hoozemans J. J. Rozemuller J. M. Veerhuis R. (2002). Neuroinflammation in Alzheimer’s disease and prion disease. *Glia* 40 232–239. 10.1002/glia.10146 12379910

[B42] Evans H. M. Howe P. R. C. Wong R. H. X. (2017). Effects of resveratrol on cognitive performance, mood and cerebrovascular function in post-menopausal women; A 14-Week randomised placebo-controlled intervention trial. *Nutrients* 9:27. 10.3390/nu9010027 28054939 PMC5295071

[B43] Farbood Y. Sarkaki A. Dianat M. Khodadadi A. Haddad M. K. Mashhadizadeh S. (2015). Ellagic acid prevents cognitive and hippocampal long-term potentiation deficits and brain inflammation in rat with traumatic brain injury. *Life Sci.* 124 120–127. 10.1016/j.lfs.2015.01.013 25637685

[B44] Farrer L. A. Cupples L. A. Haines J. L. Hyman B. Kukull W. A. Mayeux R. (1997). Effects of age, sex, and ethnicity on the association between apolipoprotein E genotype and Alzheimer disease. A meta-analysis. APOE and Alzheimer Disease Meta Analysis Consortium. *JAMA* 278 1349–1356. 10.1001/jama.1997.03550160069041 9343467

[B45] Feng J. Wang J. Du Y. Liu Y. Zhang W. Chen J. (2018). Dihydromyricetin inhibits microglial activation and neuroinflammation by suppressing NLRP3 inflammasome activation in APP/PS1 transgenic mice. *CNS Neurosci. Ther.* 10.1111/cns.12983 [Epub ahead of print]. 29869390 PMC6282966

[B46] Fleisher A. S. Raman R. Siemers E. R. Becerra L. Clark C. M. Dean R. A. (2008). Phase 2 safety trial targeting amyloid β production with a γ-secretase inhibitor in Alzheimer disease. *Arch. Neurol.* 65 1031–1038. 10.1001/archneur.65.8.1031 18695053 PMC2682361

[B47] Foti M. C. (2007). Antioxidant properties of phenols. *J. Pharm. Pharmacol.* 59 1673–1685. 10.1211/jpp.59.12.0010 18053330

[B48] Fougère B. Boulanger E. Nourhashémi F. Guyonnet S. Cesari M. (2017). Chronic inflammation: accelerator of biological aging. *J. Gerontol. A Biol. Sci. Med. Sci.* 72 1218–1225. 10.1093/gerona/glw240 28003373

[B49] Franceschi C. Bonafè M. Valensin S. Olivieri F. De Luca M. Ottaviani E. (2006). Inflamm-aging: an evolutionary perspective on immunosenescence. *Ann. N. Y. Acad. Sci.* 908 244–254. 10.1111/j.1749-6632.2000.tb06651.x 10911963

[B50] Frisardi V. Solfrizzi V. Seripa D. Capurso C. Santamato A. Sancarlo D. (2010). Metabolic-cognitive syndrome: a cross-talk between metabolic syndrome and Alzheimer’s disease. *Ageing Res. Rev.* 9 399–417. 10.1016/j.arr.2010.04.007 20444434

[B51] Galasko D. R. Peskind E. Clark C. M. Quinn J. F. Ringman J. M. Jicha G. A. (2012). Antioxidants for Alzheimer disease: a randomized clinical trial with cerebrospinal fluid biomarker measures. *Arch. Neurol.* 69 836–841. 10.1001/archneurol.2012.85 22431837 PMC3661272

[B52] Garlanda C. Dinarello C. A. Mantovani A. (2013). The Interleukin-1 family: back to the future. *Immunity* 39 1003–1018. 10.1016/j.immuni.2013.11.010 24332029 PMC3933951

[B53] Garraux G. Phillips C. Schrouff J. Kreisler A. Lemaire C. Degueldre C. (2013). Multiclass classification of FDG PET scans for the distinction between Parkinson’s disease and atypical Parkinsonian syndromes. *Neuroimage Clin.* 2 883–893. 10.1016/j.nicl.2013.06.004 24179839 PMC3778264

[B54] Gatson J. W. Liu M.-M. Abdelfattah K. Wigginton J. G. Smith S. Wolf S. (2013). Resveratrol decreases inflammation in the brain of mice with mild traumatic brain injury. *J. Trauma Acute Care Surg.* 74 470–44; discussion 474–475. 10.1097/TA.0b013e31827e1f51 23354240

[B55] Gatz M. Reynolds C. A. Fratiglioni L. Johansson B. Mortimer J. A. Berg S. (2006). Role of genes and environments for explaining Alzheimer disease. *Arch. Gen. Psychiatry* 63 168–174. 10.1001/archpsyc.63.2.168 16461860

[B56] Gavett B. E. Stern R. A. Cantu R. C. Nowinski C. J. McKee A. C. (2010). Mild traumatic brain injury: a risk factor for neurodegeneration. *Alzheimers Res. Ther.* 2:18. 10.1186/alzrt42 20587081 PMC2919698

[B57] Giasson B. I. Covy J. P. Bonini N. M. Hurtig H. I. Farrer M. J. Trojanowski J. Q. (2006). Biochemical and pathological characterization of Lrrk2. *Ann. Neurol.* 59 315–322. 10.1002/ana.20791 16437584

[B58] Gillardon F. Schmid R. Draheim H. (2012). Parkinson’s disease-linked leucine-rich repeat kinase 2(R1441G) mutation increases proinflammatory cytokine release from activated primary microglial cells and resultant neurotoxicity. *Neuroscience* 208 41–48. 10.1016/j.neuroscience.2012.02.001 22342962

[B59] Gustot A. Gallea J. I. Sarroukh R. Celej M. S. Ruysschaert J.-M. Raussens V. (2015). Amyloid fibrils are the molecular trigger of inflammation in Parkinson’s Disease. *Biochem. J.* 473 323–333. 10.1042/BJ20150617 26272943

[B60] Halle A. Hornung V. Petzold G. C. Stewart C. R. Monks B. G. Reinheckel T. (2008). The NALP3 inflammasome is involved in the innate immune response to amyloid-beta. *Nat. Immunol.* 9 857–865. 10.1038/ni.1636 18604209 PMC3101478

[B61] Hein A. M. Stasko M. R. Matousek S. B. Scott-McKean J. J. Maier S. F. Olschowka J. A. (2010). Sustained hippocampal IL-1beta overexpression impairs contextual and spatial memory in transgenic mice. *Brain Behav. Immun.* 24 243–253. 10.1016/j.bbi.2009.10.002 19825412 PMC2818290

[B62] Hendricks A. Krengel M. Iverson K. M. Kimerling R. Tun C. Amara J. (2012). “Estimating the costs of care,” in *PTSD and Mild Traumatic Brain Injury* eds Vasterling J. Bryant R. A. Keane T. M. (New York, NY: Guilford Press) 260–281.

[B63] Heneka M. T. Carson M. J. Khoury J. El Landreth G. E. Brosseron F. Feinstein D. L. (2015). Neuroinflammation in Alzheimer’s disease. *Lancet Neurol.* 14 388–405. 10.1016/S1474-4422(15)70016-5 25792098 PMC5909703

[B64] Heneka M. T. Kummer M. P. Stutz A. Delekate A. Schwartz S. Vieira-Saecker A. (2013). NLRP3 is activated in Alzheimer’s disease and contributes to pathology in APP/PS1 mice. *Nature* 493 674–678. 10.1038/nature11729 23254930 PMC3812809

[B65] Herman F. J. Pasinetti G. M. (2018). Principles of inflammasome priming and inhibition: implications for psychiatric disorders. *Brain Behav. Immun.* 10.1016/j.bbi.2018.06.010 [Epub ahead of print]. 29902514 PMC6526722

[B66] Ho L. Cheng H. Wang J. Simon J. E. Wu Q. Zhao D. (2018). A comprehensive database and analysis framework to incorporate multiscale data types and enable integrated analysis of bioactive polyphenols. *Mol. Pharm.* 15 840–850. 10.1021/acs.molpharmaceut.7b00412 28665131 PMC6016761

[B67] Ho L. Yemul S. Wang J. Pasinetti G. M. (2009). Grape seed polyphenolic extract as a potential novel therapeutic agent in tauopathies. *J. Alzheimers Dis.* 16 433–439. 10.3233/JAD-2009-0969 19221432 PMC2800939

[B68] Hogan D. B. (2014). Long-term efficacy and toxicity of cholinesterase inhibitors in the treatment of Alzheimer disease. *Can. J. Psychiatry* 59 618–623. 10.1177/070674371405901202 25702360 PMC4304580

[B69] Honig L. S. Vellas B. Woodward M. Boada M. Bullock R. Borrie M. (2018). Trial of Solanezumab for mild dementia due to Alzheimer’s Disease. *N. Engl. J. Med.* 378 321–330. 10.1056/NEJMoa1705971 29365294

[B70] Imamura K. Hishikawa N. Sawada M. Nagatsu T. Yoshida M. Hashizume Y. (2003). Distribution of major histocompatibility complex class II-positive microglia and cytokine profile of Parkinson’s disease brains. *Acta Neuropathol.* 106 518–526. 10.1007/s00401-003-0766-2 14513261

[B71] Jankovic J. (2008). Parkinson’s disease: clinical features and diagnosis. *J. Neurol. Neurosurg.* 79 368–376. 10.1136/jnnp.2007.131045 18344392

[B72] Jhang J. J. Lu C. C. Ho C. Y. Cheng Y. T. Yen G. C. (2015). Protective Effects of catechin against monosodium urate-induced inflammation through the modulation of NLRP3 inflammasome activation. *J. Agric. Food Chem.* 63 7343–7352. 10.1021/acs.jafc.5b02605 26234731

[B73] Jiang W. Huang Y. Han N. He F. Li M. Bian Z. (2016). Quercetin suppresses NLRP3 inflammasome activation and attenuates histopathology in a rat model of spinal cord injury. *Spinal Cord* 54 592–596. 10.1038/sc.2015.227 26754474

[B74] Jin F. Wu Q. Lu Y.-F. Gong Q.-H. Shi J.-S. (2008). Neuroprotective effect of resveratrol on 6-OHDA-induced Parkinson’s disease in rats. *Eur. J. Pharmacol.* 600 78–82. 10.1016/j.ejphar.2008.10.005 18940189

[B75] Jin H. Kanthasamy A. Ghosh A. Anantharam V. Kalyanaraman B. Kanthasamy A. G. (2014). Mitochondria-targeted antioxidants for treatment of Parkinson’s disease: preclinical and clinical outcomes. *Biochim. Biophys. Acta* 1842 1282–1294. 10.1016/j.bbadis.2013.09.007 24060637 PMC3961561

[B76] Johnson V. E. Stewart J. E. Begbie F. D. Trojanowski J. Q. Smith D. H. Stewart W. (2013). Inflammation and white matter degeneration persist for years after a single traumatic brain injury. *Brain* 136 28–42. 10.1093/brain/aws322 23365092 PMC3562078

[B77] Jordan D. Relkin N. R. Ravdin L. D. Jacobs A. R. Bennett A. Gandy S. (1997). Apolipoprotein chronic traumatic brain injury in boxing. *JAMA* 278 136–140. 9214529

[B78] Karch C. M. Cruchaga C. Goate A. (2014). Alzheimer’s Disease genetics: from the bench to the clinic. *Neuron* 83 11–26. 10.1016/j.neuron.2014.05.041 24991952 PMC4120741

[B79] Kenjiro O. Kazuhiro H. Hironobu N. Masahito Y. (2004). Curcumin has potent anti-amyloidogenic effects for Alzheimer’s β-amyloid fibrils in vitro. *J. Neurosci. Res.* 75 742–750. 10.1002/jnr.20025 14994335

[B80] Kent K. Charlton K. Roodenrys S. Batterham M. Potter J. Traynor V. (2017). Consumption of anthocyanin-rich cherry juice for 12 weeks improves memory and cognition in older adults with mild-to-moderate dementia. *Eur. J. Nutr.* 56 333–341. 10.1007/s00394-015-1083-y 26482148

[B81] Khan M. M. Ahmad A. Ishrat T. Khan M. B. Hoda M. N. Khuwaja G. (2010). Resveratrol attenuates 6-hydroxydopamine-induced oxidative damage and dopamine depletion in rat model of Parkinson’s disease. *Brain Res.* 1328 139–151. 10.1016/j.brainres.2010.02.031 20167206

[B82] Kim B. Yang M. S. Choi D. Kim J. H. Kim H. S. Seol W. (2012). Impaired inflammatory responses in murine lrrk2-knockdown brain microglia. *PLoS One* 7:e34693. 10.1371/journal.pone.0034693 22496842 PMC3322140

[B83] Kim J. Basak J. M. Holtzman D. M. (2009). The role of apolipoprotein E in Alzheimer’s disease. *Neuron* 63 287–303. 10.1016/j.neuron.2009.06.026 19679070 PMC3044446

[B84] Kitazawa M. Cheng D. Tsukamoto M. Koike M. Wes P. D. Vasilevko V. (2011). Blocking interleukin-1 signaling rescues cognition, attenuates tau pathology, and restores neuronal β-Catenin pathway function in an Alzheimer’s Disease model. *J. Immunol.* 187 6539–6549. 10.4049/jimmunol.1100620 22095718 PMC4072218

[B85] Klein A. M. Kowall N. W. Ferrante R. J. (1999). Neurotoxicity and oxidative damage of beta amyloid 1-42 versus beta amyloid 1-40 in the mouse cerebral cortex. *Ann. N. Y. Acad. Sci.* 893 314–320. 10672257 10.1111/j.1749-6632.1999.tb07845.x

[B86] Koda T. Kuroda Y. Imai H. (2009). Rutin supplementation in the diet has protective effects against toxicant-induced hippocampal injury by suppression of microglial activation and pro-inflammatory cytokines. *Cell. Mol. Neurobiol.* 29 523–531. 10.1007/s10571-008-9344-4 19156514 PMC11506263

[B87] Kondapalli C. Kazlauskaite A. Zhang N. Woodroof H. I. Campbell D. G. Gourlay R. (2012). PINK1 is activated by mitochondrial membrane potential depolarization and stimulates Parkin E3 ligase activity by phosphorylating Serine 65. *Open Biol.* 2:120080. 10.1098/rsob.120080 22724072 PMC3376738

[B88] Kowal S. L. Dall T. M. Chakrabarti R. Storm M. V. Jain A. (2013). The current and projected economic burden of Parkinson’s disease in the United States. *Mov. Disord.* 28 311–318. 10.1002/mds.25292 23436720

[B89] Krikorian R. Nash T. A. Shidler M. D. Shukitt-Hale B. Joseph J. A. (2010). Concord grape juice supplementation improves memory function in older adults with mild cognitive impairment. *Br. J. Nutr.* 103 730–734. 10.1017/S0007114509992364 20028599

[B90] Laura G. Ennio O. Gary W. (2004). Non-steroidal anti-inflammatory drugs (NSAIDs) in Alzheimer’s disease: old and new mechanisms of action. *J. Neurochem.* 91 521–536. 10.1111/j.1471-4159.2004.02743.x 15485484

[B91] Lee J. Torosyan N. Silverman D. H. (2017). Examining the impact of grape consumption on brain metabolism and cognitive function in patients with mild decline in cognition: a double-blinded placebo controlled pilot study. *Exp. Gerontol.* 87 121–128. 10.1016/j.exger.2016.10.004 27856335

[B92] L’Episcopo F. Tirolo C. Serapide M. F. Caniglia S. Testa N. Leggio L. (2018). Microglia polarization, gene-environment interactions and Wnt/β-Catenin Signaling: emerging roles of Glia-Neuron and Glia-Stem/Neuroprogenitor crosstalk for dopaminergic neurorestoration in aged Parkinsonian brain. *Front. Aging Neurosci.* 10:12. 10.3389/fnagi.2018.00012 29483868 PMC5816064

[B93] Leyns C. E. G. Ulrich J. D. Finn M. B. Stewart F. R. Koscal L. J. Remolina Serrano J. (2017). TREM2 deficiency attenuates neuroinflammation and protects against neurodegeneration in a mouse model of tauopathy. *Proc. Natl. Acad. Sci. U.S.A.* 114 11524–11529. 10.1073/pnas.1710311114 29073081 PMC5663386

[B94] Lian Q. Nie Y. Zhang X. Tan B. Cao H. Chen W. (2016). Effects of grape seed proanthocyanidin on Alzheimer’s disease in vitro and in vivo. *Exp. Ther. Med.* 12 1681–1692. 10.3892/etm.2016.3530 27588088 PMC4998082

[B95] Liang Y. Jing X. Zeng Z. Bi W. Chen Y. Wu X. (2015). Rifampicin attenuates rotenone-induced inflammation via suppressing NLRP3 inflammasome activation in microglia. *Brain Res.* 1622 43–50. 10.1016/j.brainres.2015.06.008 26086368

[B96] Lionaki E. Markaki M. Palikaras K. Tavernarakis N. (2015). Mitochondria, autophagy and age-associated neurodegenerative diseases: new insights into a complex interplay. *Biochim. Biophys. Acta* 1847 1412–1423. 10.1016/j.bbabio.2015.04.010 25917894

[B97] Lista S. Hampel H. (2017). Synaptic degeneration and neurogranin in the pathophysiology of Alzheimer’s disease. *Expert Rev. Neurother.* 17 47–57. 10.1080/14737175.2016.1204234 27332958

[B98] Liu A. K. L. Chang R. C.-C. Pearce R. K. B. Gentleman S. M. (2015). Nucleus basalis of Meynert revisited: anatomy, history and differential involvement in Alzheimer’s and Parkinson’s disease. *Acta Neuropathol.* 129 527–540. 10.1007/s00401-015-1392-5 25633602 PMC4366544

[B99] Liu H. J. Pan X. X. Liu B. Q. Gui X. Hu L. Jiang C. Y. (2017). Grape seed-derived procyanidins alleviate gout pain via NLRP3 inflammasome suppression. *J. Neuroinflammation* 14:74. 10.1186/s12974-017-0849-y 28376889 PMC5381065

[B100] Liu W. Liu X. Li Y. Zhao J. Liu Z. Hu Z. (2017). LRRK2 promotes the activation of NLRC4 inflammasome during *Salmonella* Typhimurium infection. *J. Exp. Med.* 214 3051–3066. 10.1084/jem.20170014 28821568 PMC5626397

[B101] Liu Y. Pukala T. L. Musgrave I. F. Williams D. M. Dehle F. C. Carver J. A. (2013). Gallic acid is the major component of grape seed extract that inhibits amyloid fibril formation. *Bioorg. Med. Chem. Lett.* 23 6336–6340. 10.1016/j.bmcl.2013.09.071 24157371

[B102] Liu Y. Qin L. Li G. Zhang W. An L. Liu B. (2003). Dextromethorphan protects dopaminergic neurons against inflammation-mediated degeneration through inhibition of microglial activation. *J. Pharmacol. Exp. Ther.* 305 212–218. 12649371 10.1124/jpet.102.043166

[B103] Long J. Gao H. Sun L. Liu J. Zhao-Wilson X. (2009). Grape extract protects mitochondria from oxidative damage and improves locomotor dysfunction and extends lifespan in a Drosophila Parkinson’s Disease Model. *Rejuvenation Res.* 12 321–331. 10.1089/rej.2009.0877 19929256

[B104] López-Otín C. Blasco M. A. Partridge L. Serrano M. Kroemer G. (2013). The hallmarks of aging. *Cell* 153 1194–1217. 10.1016/j.cell.2013.05.039 23746838 PMC3836174

[B105] Lovell M. A. Xie C. Markesbery W. R. (2001). Acrolein is increased in Alzheimer’s disease brain and is toxic to primary hippocampal cultures. *Neurobiol. Aging* 22 187–194. 10.1016/S0197-4580(00)00235-9 11182468

[B106] Lu M. Sun X.-L. Qiao C. Liu Y. Ding J.-H. Hu G. (2014). Uncoupling protein 2 deficiency aggravates astrocytic endoplasmic reticulum stress and nod-like receptor protein 3 inflammasome activation. *Neurobiol. Aging* 35 421–430. 10.1016/j.neurobiolaging.2013.08.015 24041971

[B107] Lyketsos C. G. Carrillo M. C. Ryan J. M. Khachaturian A. S. Trzepacz P. Amatniek J. (2011). Neuropsychiatric symptoms in Alzheimer’s disease. *Alzheimers Dement.* 7 532–539. 10.1016/j.jalz.2011.05.2410 21889116 PMC3299979

[B108] Magalingam K. B. Radhakrishnan A. Ping N. S. Haleagrahara N. (2018). Current concepts of neurodegenerative mechanisms in Alzheimer’s Disease. *Biomed. Res. Int.* 2018:3740461. 10.1155/2018/3740461 29707568 PMC5863339

[B109] Mao Z. Liu C. Ji S. Yang Q. Ye H. Han H. (2017). The NLRP3 inflammasome is involved in the pathogenesis of Parkinson’s disease in rats. *Neurochem. Res.* 42 1104–1115. 10.1007/s11064-017-2185-0 28247334

[B110] Marambaud P. Zhao H. Davies P. (2005). Resveratrol promotes clearance of Alzheimer’s disease amyloid-beta peptides. *J. Biol. Chem.* 280 37377–37382. 10.1074/jbc.M508246200 16162502

[B111] Mayo Foundation for Medical Education and Research [MFMER] (2017). Parkinson’s Disease. *Natl. Inst. Aging* 386 896–912.

[B112] McCann H. Stevens C. H. Cartwright H. Halliday G. M. (2014). Synucleinopathy phenotypes. *Parkinsonism Relat. Disord.* 20 S62–S67. 10.1016/S1353-8020(13)70017-8 24262191

[B113] McCormack A. L. Thiruchelvam M. Manning-Bog A. B. Thiffault C. Langston J. W. Cory-Slechta D. A. (2002). Environmental risk factors and Parkinson’s disease: selective degeneration of nigral dopaminergic neurons caused by the herbicide paraquat. *Neurobiol. Dis.* 10 119–127. 10.1006/nbdi.2002.0507 12127150

[B114] McGeer P. L. Itagaki S. Boyes B. E. McGeer E. G. (1988). Reactive microglia are positive for HLA-DR in the substantia nigra of Parkinson’s and Alzheimer’s disease brains. *Neurology* 38 1285–1291. 3399080 10.1212/wnl.38.8.1285

[B115] McKee A. C. Cantu R. C. Nowinski C. J. Hedley-Whyte E. T. Gavett B. E. Budson A. E. (2009). Chronic traumatic encephalopathy in athletes: progressive tauopathy after repetitive head injury. *J. Neuropathol. Exp. Neurol.* 68 709–735. 10.1097/NEN.0b013e3181a9d503 19535999 PMC2945234

[B116] McKee A. C. Gavett B. E. Stern R. A. Nowinski C. J. Cantu R. C. Kowall N. W. (2010). TDP-43 proteinopathy and motor neuron disease in chronic traumatic encephalopathy. *J. Neuropathol. Exp. Neurol.* 69 918–929. 10.1097/NEN.0b013e3181ee7d85 20720505 PMC2951281

[B117] McKee A. C. Stern R. A. Nowinski C. J. Stein T. D. Alvarez V. E. Daneshvar D. H. (2013). The spectrum of disease in chronic traumatic encephalopathy. *Brain* 136 43–64. 10.1093/brain/aws307 23208308 PMC3624697

[B118] Mercer L. D. Kelly B. L. Horne M. K. Beart P. M. (2005). Dietary polyphenols protect dopamine neurons from oxidative insults and apoptosis: investigations in primary rat mesencephalic cultures. *Biochem. Pharmacol.* 69 339–345. 10.1016/j.bcp.2004.09.018 15627486

[B119] Michaelson D. M. (2014). APOE 𝜀4: the most prevalent yet understudied risk factor for Alzheimer’s disease. *Alzheimer’s Dement.* 10 861–868. 10.1016/j.jalz.2014.06.015 25217293

[B120] Misawa T. Saitoh T. Kozaki T. Park S. Takahama M. Akira S. (2015). Resveratrol inhibits the acetylated alpha-tubulin-mediated assembly of the NLRP3-inflammasome. *Int. Immunol.* 27 425–434. 10.1093/intimm/dxv018 25855661

[B121] Mittal K. Mani R. J. Katare D. P. (2016). Type 3 diabetes: cross talk between differentially regulated proteins of type 2 Diabetes Mellitus and Alzheimer’s Disease. *Sci. Rep.* 6:25589. 10.1038/srep25589 27151376 PMC4858691

[B122] Mi-Jeong K. Ah-Reum S. Jung-Yoon Y. Cheng-Hao J. Yoo-Hyun L. Jun K. Y. (2011). Gallic acid, a histone acetyltransferase inhibitor, suppresses β-amyloid neurotoxicity by inhibiting microglial-mediated neuroinflammation. *Mol. Nutr. Food Res.* 55 1798–1808. 10.1002/mnfr.201100262 22038937

[B123] Mo J.-J. Liu L.-Y. Peng W.-B. Rao J. Liu Z. Cui L.-L. (2017). The effectiveness of creatine treatment for Parkinson’s disease: an updated meta-analysis of randomized controlled trials. *BMC Neurol.* 17:105. 10.1186/s12883-017-0885-3 28577542 PMC5457735

[B124] Montagne A. Barnes S. R. Sweeney M. D. Halliday M. R. Sagare A. P. Zhao Z. (2015). Blood-Brain barrier breakdown in the aging human hippocampus. *Neuron* 85 296–302. 10.1016/j.neuron.2014.12.032 25611508 PMC4350773

[B125] Moussa C. Hebron M. Huang X. Ahn J. Rissman R. A. Aisen P. S. (2017). Resveratrol regulates neuro-inflammation and induces adaptive immunity in Alzheimer’s disease. *J. Neuroinflammation* 14:1. 10.1186/s12974-016-0779-0 28086917 PMC5234138

[B126] Mrak R. E. Griffin W. S. (2001). Interleukin-1, neuroinflammation, and Alzheimer’s disease. *Neurobiol. Aging* 22 903–908. 11754997 10.1016/s0197-4580(01)00287-1

[B127] National Hospital Discharge Survey [NHDS] (2010). *National Hospital Ambulatory Medical Care Survey (NHAMCS), 2010 and National Vital Statistics System (NVSS), 2010.* Atlanta, GA: Centers for Disease Control and Prevention.

[B128] Neuroimaging I. F. Kaga K. van der Knaap M. S. Valk J. Greene J. Bone I. (2010). Atlas of epilepsies. *DeJong’s Neurol. Exam.* 111 1–4. 10.1007/978-1-84882-128-6

[B129] Neus M. Noemi G. Montserrat P. Anna A. Mayte B. (2014). Procyanidin B2 inhibits inflammasome-mediated IL-1β production in lipopolysaccharide-stimulated macrophages. *Mol. Nutr. Food Res.* 59 262–269. 10.1002/mnfr.201400370 25379992

[B130] Ninds Net-Pd Investigators (2008). A pilot clinical trial of creatine and minocycline in early Parkinson disease: 18-month results. *Clin. Neuropharmacol.* 31 141–150. 10.1097/WNF.0b013e3181342f32 18520981 PMC4372145

[B131] Nurmikko T. J. Serpell M. G. Hoggart B. Toomey P. J. Morlion B. J. Haines D. (2007). Sativex successfully treats neuropathic pain characterised by allodynia: a randomised, double-blind, placebo-controlled clinical trial. *Pain* 133 210–220. 10.1016/j.pain.2007.08.028 17997224

[B132] O’Brien J. T. Markus H. S. (2014). Vascular risk factors and Alzheimer’s disease. *BMC Med.* 12:218. 10.1186/s12916-014-0218-y 25385509 PMC4226870

[B133] Ofengeim D. Mazzitelli S. Ito Y. DeWitt J. P. Mifflin L. Zou C. (2017). RIPK1 mediates a disease-associated microglial response in Alzheimer’s disease. *Proc. Natl. Acad. Sci. U.S.A.* 114 E8788–E8797. 10.1073/pnas.1714175114 28904096 PMC5642727

[B134] Ojala J. Alafuzoff I. Herukka S.-K. van Groen T. Tanila H. Pirttila T. (2009). Expression of interleukin-18 is increased in the brains of Alzheimer’s disease patients. *Neurobiol. Aging* 30 198–209. 10.1016/j.neurobiolaging.2007.06.006 17658666

[B135] Olanow C. W. Schapira A. H. (2013). Therapeutic prospects for Parkinson disease. *Ann. Neurol.* 74 337–347. 10.1002/ana.24011 24038341

[B136] Omalu B. (2014). Chronic traumatic encephalopathy. *Prog. Neurol. Surg.* 28 38–49. 10.1159/000358761 24923391

[B137] Ono K. Condron M. M. Ho L. Wang J. Zhao W. Pasinetti G. M. (2008). Effects of grape seed-derived polyphenols on amyloid beta-protein self-assembly and cytotoxicity. *J. Biol. Chem.* 283 32176–32187. 10.1074/jbc.M806154200 18815129 PMC2583320

[B138] O’Sullivan S. S. Williams D. R. Gallagher D. A. Massey L. A. Silveira-Moriyama L. Lees A. J. (2008). Nonmotor symptoms as presenting complaints in Parkinson’s disease: a clinicopathological study. *Mov. Disord.* 23 101–106. 10.1002/mds.21813 17994582

[B139] Pasinetti G. M. Wang J. Ho L. Zhao W. Dubner L. (2015). Roles of resveratrol and other grape-derived polyphenols in Alzheimer’s disease prevention and treatment. *Biochim. Biophys. Acta* 1852 1202–1208. 10.1016/j.bbadis.2014.10.006 25315300 PMC4380832

[B140] Pasqualetti P. Bonomini C. Dal Forno G. Paulon L. Sinforiani E. Marra C. (2009). A randomized controlled study on effects of ibuprofen on cognitive progression of Alzheimer’s disease. *Aging Clin. Exp. Res.* 21 102–110. 19448381 10.1007/BF03325217

[B141] Pawelec G. (2017). Immunosenescence and cancer. *Biogerontology* 18 717–721. 10.1007/s10522-017-9682-z 28220304

[B142] Perron N. R. Brumaghim J. L. (2009). A review of the antioxidant mechanisms of polyphenol compounds related to iron binding. *Cell Biochem. Biophys.* 53 75–100. 10.1007/s12013-009-9043-x 19184542

[B143] Pezzuto J. M. (2008). Grapes and human health: a perspective. *J. Agric. Food Chem.* 56 6777–6784. 10.1021/jf800898p 18662007

[B144] Pfleger C. M. Wang J. Friedman L. Vittorino R. Conley L. M. Ho L. (2010). Grape-seed polyphenolic extract improves the eye phenotype in a Drosophila model of tauopathy. *Int. J. Alzheimers Dis.* 2010:576357. 10.4061/2010/576357 20871666 PMC2943075

[B145] Pistollato F. Iglesias R. C. Ruiz R. Aparicio S. Crespo J. Lopez L. D. (2018). Nutritional patterns associated with the maintenance of neurocognitive functions and the risk of dementia and Alzheimer’s disease: a focus on human studies. *Pharmacol. Res.* 131 32–43. 10.1016/j.phrs.2018.03.012 29555333

[B146] Poewe W. Antonini A. Zijlmans J. C. M. Burkhard P. R. Vingerhoets F. (2010). Levodopa in the treatment of Parkinson’s disease: an old drug still going strong. *Clin. Interv. Aging* 5 229–238. 20852670 10.2147/cia.s6456 PMC2938030

[B147] Pottier C. Wallon D. Rousseau S. Rovelet-Lecrux A. Richard A.-C. Rollin-Sillaire A. (2013). TREM2 R47H variant as a risk factor for early-onset Alzheimer’s disease. *J. Alzheimers Dis.* 35 45–49. 10.3233/JAD-122311 23380991

[B148] Przedborski S. Vila M. Jackson-Lewis V. (2003). Neurodegeneration: what is it and where are we? *J. Clin. Invest.* 111 3–10. 10.1172/JCI17522 12511579 PMC151843

[B149] Rajamaki K. Lappalainen J. Oorni K. Valimaki E. Matikainen S. Kovanen P. T. (2010). Cholesterol crystals activate the NLRP3 inflammasome in human macrophages: a novel link between cholesterol metabolism and inflammation. *PLoS One* 5:e11765. 10.1371/journal.pone.0011765 20668705 PMC2909263

[B150] Ramlackhansingh A. F. Brooks D. J. Greenwood R. J. Bose S. K. Turkheimer F. E. Kinnunen K. M. (2011). Inflammation after trauma: microglial activation and traumatic brain injury. *Ann. Neurol.* 70 374–383. 10.1002/ana.22455 21710619

[B151] Reger M. A. Watson G. S. Green P. S. Wilkinson C. W. Baker L. D. Cholerton B. (2008). Intranasal insulin improves cognition and modulates β-amyloid in early AD. *Neurology* 70 440–448. 17942819 10.1212/01.WNL.0000265401.62434.36

[B152] Rho K. A. Kim M. K. (2006). Effects of different grape formulations on antioxidative capacity, lipid peroxidation and oxidative DNA damage in aged rats. *J. Nutr. Sci. Vitaminol.* 52 33–46. 10.3177/jnsv.52.33 16637228

[B153] Rice-evans C. A. Miller N. J. Bolwell P. G. Bramley P. M. Pridham J. B. (1995). The relative antioxidant activities of plant-derived polyphenolic flavonoids. *Free Radic. Res.* 22 375–383. 10.3109/10715769509145649 7633567

[B154] Rouhiainen A. Kuja-Panula J. Wilkman E. Pakkanen J. Stenfors J. Tuominen R. K. (2004). Regulation of monocyte migration by amphoterin (HMGB1). *Blood* 104 1174–1182. 10.1182/blood-2003-10-3536 15130941

[B155] Ruiz-Miyazawa K. W. Staurengo-Ferrari L. Mizokami S. S. Domiciano T. P. Vicentini F. T. M. C. Camilios-Neto D. (2017). Quercetin inhibits gout arthritis in mice: induction of an opioid-dependent regulation of inflammasome. *Inflammopharmacology* 25 555–570. 10.1007/s10787-017-0356-x 28508104

[B156] Saijo K. Crotti A. Glass C. K. (2010). “Chapter 2 - nuclear receptors, inflammation, and neurodegenerative diseases,” in *Advances in Immunology* ed. Frederick W. (Cambridge, MA: Academic Press) 21–59. 10.1016/S0065-2776(10)06002-5 20728023

[B157] Santa-Maria I. Diaz-Ruiz C. Ksiezak-Reding H. Chen A. Ho L. Wang J. (2012). GSPE interferes with tau aggregation in vivo: implication for treating tauopathy. *Neurobiol. Aging* 33 2072–2081. 10.1016/j.neurobiolaging.2011.09.027 22054871 PMC3472512

[B158] Sarkaki A. Eidypour Z. Motamedi F. Keramati K. Farbood Y. (2012). Motor disturbances and thalamic electrical power of frequency bands’ improve by grape seed extract in animal model of Parkinson’s disease. *Avicenna J. Phytomed.* 2 222–232. 25050252 PMC4075680

[B159] Sawda C. Moussa C. Turner R. S. (2017). Resveratrol for Alzheimer’s disease. *Ann. N. Y. Acad. Sci.* 1403 142–149. 10.1111/nyas.13431 28815614 PMC5664214

[B160] Sayre L. M. Zelasko D. A. Harris P. L. Perry G. Salomon R. G. Smith M. A. (2002). 4-Hydroxynonenal-Derived advanced lipid peroxidation end products are increased in Alzheimer’s Disease. *J. Neurochem.* 68 2092–2097. 10.1046/j.1471-4159.1997.68052092.x 9109537

[B161] Schmidt M. Zhukareva V. Newell K. Lee V. Trojanowski J. (2001). Tau isoform profile and phosphorylation state in dementia pugilistica recapitulate Alzheimer’s disease. *Acta Neuropathol.* 101 518–524. 10.1007/s004010000330 11484824

[B162] Schmitz T. W. Nathan Spreng R. (2016). Basal forebrain degeneration precedes and predicts the cortical spread of Alzheimer’s pathology. *Nat. Commun.* 7:13249. 10.1038/ncomms13249 27811848 PMC5097157

[B163] Schültke E. Kamencic H. Zhao M. Tian G.-F. Baker A. J. Griebel R. W. (2005). Neuroprotection following fluid percussion brain trauma: a pilot study using quercetin. *J. Neurotrauma* 22 1475–1484. 10.1089/neu.2005.22.1475 16379584

[B164] Sheng Z. Oka S. Tsuchimoto D. Abolhassani N. Nomaru H. Sakumi K. (2012). 8-Oxoguanine causes neurodegeneration during MUTYH-mediated DNA base excision repair. *J. Clin. Invest.* 122 4344–4361. 10.1172/JCI65053 23143307 PMC3533558

[B165] Simard A. R. Soulet D. Gowing G. Julien J.-P. Rivest S. (2006). Bone marrow-derived microglia play a critical role in restricting senile plaque formation in Alzheimer’s Disease. *Neuron* 49 489–502. 10.1016/j.neuron.2006.01.022 16476660

[B166] Simon E. Obst J. Gomez-Nicola D. (2018). The Evolving dialogue of microglia and neurons in Alzheimer’s Disease: microglia as necessary transducers of pathology. *Neuroscience* 10.1016/j.neuroscience.2018.01.059 [Epub ahead of print]. 29427657

[B167] Sims R. van der Lee S. J. Naj A. C. Bellenguez C. Badarinarayan N. Jakobsdottir J. (2017). Rare coding variants in PLCG2, ABI3, and TREM2 implicate microglial-mediated innate immunity in Alzheimer’s disease. *Nat. Genet.* 49 1373–1384. 10.1038/ng.3916 28714976 PMC5669039

[B168] Singh R. Meier T. B. Kuplicki R. Savitz J. Mukai I. Cavanagh L. (2014). Relationship of collegiate football experience and concussion with hippocampal volume and cognitive outcomes. *JAMA* 311 1883–1888. 10.1001/jama.2014.3313 24825643

[B169] Small G. W. Kepe V. Siddarth P. Ercoli L. M. Merrill D. A. Donoghue N. (2013). PET scanning of brain tau in retired national football league players: preliminary findings. *Am. J. Geriatr. Psychiatry* 21 138–144. 10.1016/j.jagp.2012.11.019 23343487

[B170] Snitz B. E. O’Meara E. S. Carlson M. C. (2009). Ginkgo biloba for preventing cognitive decline in older adults: a randomized trial. *JAMA* 302 2663–2670. 10.1001/jama.2009.1913 20040554 PMC2832285

[B171] Soininen H. West C. Robbins J. Niculescu L. (2007). Long-term efficacy and safety of celecoxib in Alzheimer’s disease. *Dement. Geriatr. Cogn. Disord.* 23 8–21. 10.1159/000096588 17068392

[B172] Solanki N. Alkadhi I. Atrooz F. Patki G. Salim S. (2015). Grape powder prevents cognitive, behavioral, and biochemical impairments in a rat model of posttraumatic stress disorder. *Nutr. Res.* 35 65–75. 10.1016/j.nutres.2014.11.008 25533441 PMC4329242

[B173] Solfrizzi V. Panza F. Colacicco A. M. D’Introno A. Capurso C. Torres F. (2004). Vascular risk factors, incidence of MCI, and rates of progression to dementia. *Neurology* 63 1882–1891. 10.1212/01.WNL.0000144281.38555.E3 15557506

[B174] Solito E. Sastre M. (2012). Microglia function in Alzheimer’s Disease. *Front. Pharmacol.* 3:14 10.3389/fphar.2012.00014 PMC3277080 22363284

[B175] Steinberg S. Stefansson H. Jonsson T. Johannsdottir H. Ingason A. Helgason H. (2015). Loss-of-function variants in ABCA7 confer risk of Alzheimer’s disease. *Nat. Genet.* 47 445–447. 10.1038/ng.3246 25807283

[B176] Stern R. A. Riley D. O. Daneshvar D. H. Nowinski C. J. Cantu R. C. McKee A. C. (2011). Long-term consequences of repetitive brain trauma: chronic traumatic encephalopathy. *PM R* 3 S460–S467. 10.1016/j.pmrj.2011.08.008 22035690

[B177] Strathearn K. E. Yousef G. G. Grace M. H. Roy S. L. Tambe M. A. Ferruzzi M. G. (2014). Neuroprotective effects of anthocyanin- and proanthocyanidin-rich extracts in cellular models of Parkinson’s disease. *Brain Res.* 1555 60–77. 10.1016/j.brainres.2014.01.047 24502982 PMC4024464

[B178] Sundström A. Nilsson L.-G. Cruts M. Adolfsson R. Van Broeckhoven C. Nyberg L. (2007). Increased risk of dementia following mild head injury for carriers but not for non-carriers of the APOE epsilon4 allele. *Int. Psychogeriatr.* 19 159–165. 10.1017/S1041610206003498 16684399

[B179] Sutterwala F. S. Haasken S. Cassel S. L. (2014). Mechanism of NLRP3 inflammasome activation. *Ann. N. Y. Acad. Sci.* 1319 82–95. 10.1111/nyas.12458 24840700 PMC4074217

[B180] Sweeney M. D. Sagare A. P. Zlokovic B. V. (2018). Blood-brain barrier breakdown in Alzheimer disease and other neurodegenerative disorders. *Nat. Rev. Neurol.* 14 133–150. 10.1038/nrneurol.2017.188 29377008 PMC5829048

[B181] Takahashi R. Ono K. Takamura Y. Mizuguchi M. Ikeda T. Nishijo H. (2015). Phenolic compounds prevent the oligomerization of alpha-synuclein and reduce synaptic toxicity. *J. Neurochem.* 134 943–955. 10.1111/jnc.13180 26016728

[B182] Tan M.-S. Yu J.-T. Jiang T. Zhu X.-C. Tan L. (2013). The NLRP3 inflammasome in Alzheimer’s Disease. *Mol. Neurobiol.* 48 875–882. 10.1007/s12035-013-8475-x 23686772

[B183] Tarkowski E. Andreasen N. Tarkowski A. Blennow K. (2003). Intrathecal inflammation precedes development of Alzheimer’s disease. *J. Neurol. Neurosurg. Psychiatry* 74 1200–1205. 10.1136/jnnp.74.9.1200 12933918 PMC1738668

[B184] Thal L. J. Ferris S. H. Kirby L. Block G. A. Lines C. R. Yuen E. (2005). A randomized, double-blind, study of rofecoxib in patients with mild cognitive impairment. *Neuropsychopharmacology* 30 1204–1215. 10.1038/sj.npp.1300690 15742005

[B185] Thomas B. Beal M. F. (2007). Parkinson’s disease. *Hum. Mol. Genet.* 16 R183–R194. 10.1093/hmg/ddm159 17911161

[B186] Traustadottir T. Davies S. S. Stock A. A. Su Y. Heward C. B. Roberts L. J. II (2009). Tart cherry juice decreases oxidative stress in healthy older men and women. *J. Nutr.* 139 1896–1900. 10.3945/jn.109.111716 19692530 PMC3151016

[B187] Turner R. S. Thomas R. G. Craft S. van Dyck C. H. Mintzer J. Reynolds B. A. (2015). A randomized, double-blind, placebo-controlled trial of resveratrol for Alzheimer disease. *Neurology* 85 1383–1391. 10.1212/WNL.0000000000002035 26362286 PMC4626244

[B188] Umeno A. Biju V. Yoshida Y. (2017). In vivo ROS production and use of oxidative stress-derived biomarkers to detect the onset of diseases such as Alzheimer’s disease. Parkinson’s disease, and diabetes. *Free Radic. Res.* 51 413–427. 10.1080/10715762.2017.1315114 28372523

[B189] Valera E. Masliah E. (2016). “Immunotherapy against α-Synuclein pathology BT - immunotherapy and biomarkers,” in *Neurodegenerative Disorders* eds Ingelsson M. Lannfelt L. (New York, NY: Springer) 63–72. 10.1007/978-1-4939-3560-4_5

[B190] Van Cauwenberghe C. Van Broeckhoven C. Sleegers K. (2016). The genetic landscape of Alzheimer disease: clinical implications and perspectives. *Genet. Med.* 18 421–430. 10.1038/gim.2015.117 26312828 PMC4857183

[B191] Verdile G. Fuller S. J. Martins R. N. (2015). The role of type 2 diabetes in neurodegeneration. *Neurobiol. Dis.* 84 22–38. 10.1016/j.nbd.2015.04.008 25926349

[B192] Wang J. Ho L. Zhao W. Ono K. Rosensweig C. Chen L. (2008). Grape-Derived polyphenolics prevent Aβ oligomerization and attenuate cognitive deterioration in a mouse model of Alzheimer’s Disease. *J. Neurosci.* 28 6388–6392. 10.1523/JNEUROSCI.0364-08.2008 18562609 PMC2806059

[B193] Wang J. Santa-Maria I. Ho L. Ksiezak-Reding H. Ono K. Teplow D. B. (2010). Grape derived polyphenols attenuate tau neuropathology in a mouse model of Alzheimer’s disease. *J. Alzheimers Dis.* 22 653–661. 10.3233/JAD-2010-101074 20858961

[B194] Wang W. Wang C. Ding X. Q. Pan Y. Gu T. T. Wang M. X. (2013). Quercetin and allopurinol reduce liver thioredoxin-interacting protein to alleviate inflammation and lipid accumulation in diabetic rats. *Br. J. Pharmacol.* 169 1352–1371. 10.1111/bph.12226 23647015 PMC3831713

[B195] Whitehouse P. J. Price D. L. Struble R. G. Clark A. W. Coyle J. T. Delon M. R. (1982). Alzheimer’s disease and senile dementia: loss of neurons in the basal forebrain. *Science* 215 1237–1239. 10.1126/science.7058341 7058341

[B196] Wu D. C. Jackson-Lewis V. Vila M. Tieu K. Teismann P. Vadseth C. (2002). Blockade of microglial activation is neuroprotective in the 1-methyl-4-phenyl-1,2,3,6-tetrahydropyridine mouse model of Parkinson disease. *J. Neurosci.* 22 1763–1771. 10.1523/JNEUROSCI.22-05-01763.2002 11880505 PMC6758858

[B197] Xu Y. Li S. Chen R. Li G. Barish P. A. You W. (2010). Antidepressant-like effect of low molecular proanthocyanidin in mice: involvement of monoaminergic system. *Pharmacol. Biochem. Behav.* 94 447–453. 10.1016/j.pbb.2009.10.007 19857512

[B198] Xue J. Yu C. Sheng W. Zhu W. Luo J. Zhang Q. (2017). The Nrf2/GCH1/BH4 axis ameliorates radiation-induced skin injury by modulating the ROS cascade. *J. Invest. Dermatol.* 137 2059–2068. 10.1016/j.jid.2017.05.019 28596000

[B199] Yan Y. Jiang W. Liu L. Wang X. Ding C. Tian Z. (2015). Dopamine controls systemic inflammation through inhibition of NLRP3 inflammasome. *Cell* 160 62–73. 10.1016/j.cell.2014.11.047 25594175

[B200] Yang H. Xiao L. Yuan Y. Luo X. Jiang M. Ni J. (2014). Procyanidin B2 inhibits NLRP3 inflammasome activation in human vascular endothelial cells. *Biochem. Pharmacol.* 92 599–606. 10.1016/j.bcp.2014.10.001 25450671

[B201] Yang L. Calingasan N. Y. Wille E. J. Cormier K. Smith K. Ferrante R. J. (2009). Combination therapy with Coenzyme Q10 and creatine produces additive neuroprotective effects in models of Parkinson’s and Huntington’s Diseases. *J. Neurochem.* 109 1427–1439. 10.1111/j.1471-4159.2009.06074.x 19476553 PMC2866530

[B202] Yang Y. Zhang X. Xu M. Wu X. Zhao F. Zhao C. (2018). Quercetin attenuates collagen-induced arthritis by restoration of Th17/Treg balance and activation of Heme Oxygenase 1-mediated anti-inflammatory effect. *Int. Immunopharmacol.* 54 153–162. 10.1016/j.intimp.2017.11.013 29149703

[B203] Zhang F. Shi J.-S. Zhou H. Wilson B. C. Hong J.-S. Gao H.-M. (2010). Resveratrol protects dopamine neurons against lipopolysaccharide-induced neurotoxicity through its anti-inflammatory actions. *Mol. Pharmacol.* 78 466–477. 10.1124/mol.110.064535 20554604 PMC2939485

[B204] Zhang P. Shao X.-Y. Qi G.-J. Chen Q. Bu L.-L. Chen L.-J. (2016). Cdk5-dependent activation of neuronal inflammasomes in Parkinson’s Disease. *Mov. Disord.* 31 366–376. 10.1002/mds.26488 26853432

[B205] Zhang X. Wu Q. Zhang Q. Lu Y. Liu J. Li W. (2017). Resveratrol attenuates early brain injury after experimental subarachnoid hemorrhage via Inhibition of NLRP3 Inflammasome activation. *Front. Neurosci.* 11:611. 10.3389/fnins.2017.00611 29163015 PMC5675880

[B206] Zhao H. Wang Q. Cheng X. Li X. Li N. Liu T. (2018). Inhibitive effect of resveratrol on the inflammation in cultured astrocytes and microglia induced by Abeta1-42. *Neuroscience* 379 390–404. 10.1016/j.neuroscience.2018.03.047 29627302

[B207] Zhao H. F. Li N. Wang Q. Cheng X. J. Li X. M. Liu T. T. (2015). Resveratrol decreases the insoluble Aβ1–42 level in hippocampus and protects the integrity of the blood–brain barrier in AD rats. *Neuroscience* 310 641–649. 10.1016/j.neuroscience.2015.10.006 26454022

[B208] Zhou R. Yazdi A. S. Menu P. Tschopp J. (2011). A role for mitochondria in NLRP3 inflammasome activation. *Nature* 469 221–225. 10.1038/nature09663 21124315

[B209] Zou P. Liu X. Li G. Wang Y. (2018). Resveratrol pretreatment attenuates traumatic brain injury in rats by suppressing NLRP3 inflammasome activation via SIRT1. *Mol. Med. Rep.* 17 3212–3217. 10.3892/mmr.2017.8241 29257276

